# Dietary cysteine and methionine promote peroxisome elevation and fat loss by induction of *CG33474* expression in *Drosophila* adipose tissue

**DOI:** 10.1007/s00018-024-05226-y

**Published:** 2024-04-22

**Authors:** Meng Liu, Li He

**Affiliations:** The First Affiliated Hospital of USTC, Division of Life Sciences and Medicine, University of Science and Technology of China, Hefei, 230027 China

**Keywords:** High-protein diet, Peroxisome, Cysteine, Methionine, *CG33474*, *PEX11G*, Fat loss

## Abstract

The high-protein diet (HPD) has emerged as a potent dietary approach to curb obesity. Peroxisome, a highly malleable organelle, adapts to nutritional changes to maintain homeostasis by remodeling its structure, composition, and quantity. However, the impact of HPD on peroxisomes and the underlying mechanism remains elusive. Using *Drosophila melanogaster* as a model system, we discovered that HPD specifically increases peroxisome levels within the adipose tissues. This HPD-induced peroxisome elevation is attributed to cysteine and methionine by triggering the expression of *CG33474*, a fly homolog of mammalian *PEX11G*. Both the overexpression of *Drosophila CG33474* and human *PEX11G* result in increased peroxisome size. In addition, cysteine and methionine diets both reduce lipid contents, a process that depends on the presence of *CG33474*. Furthermore, *CG33474* stimulates the breakdown of neutral lipids in a cell-autonomous manner. Moreover, the expression of *CG33474* triggered by cysteine and methionine requires TOR signaling. Finally, we found that *CG33474* promotes inter-organelle contacts between peroxisomes and lipid droplets (LDs), which might be a potential mechanism for *CG33474*-induced fat loss. In summary, our findings demonstrate that *CG33474/PEX11G* may serve as an essential molecular bridge linking HPD to peroxisome dynamics and lipid metabolism.

**Graphical abstract:**

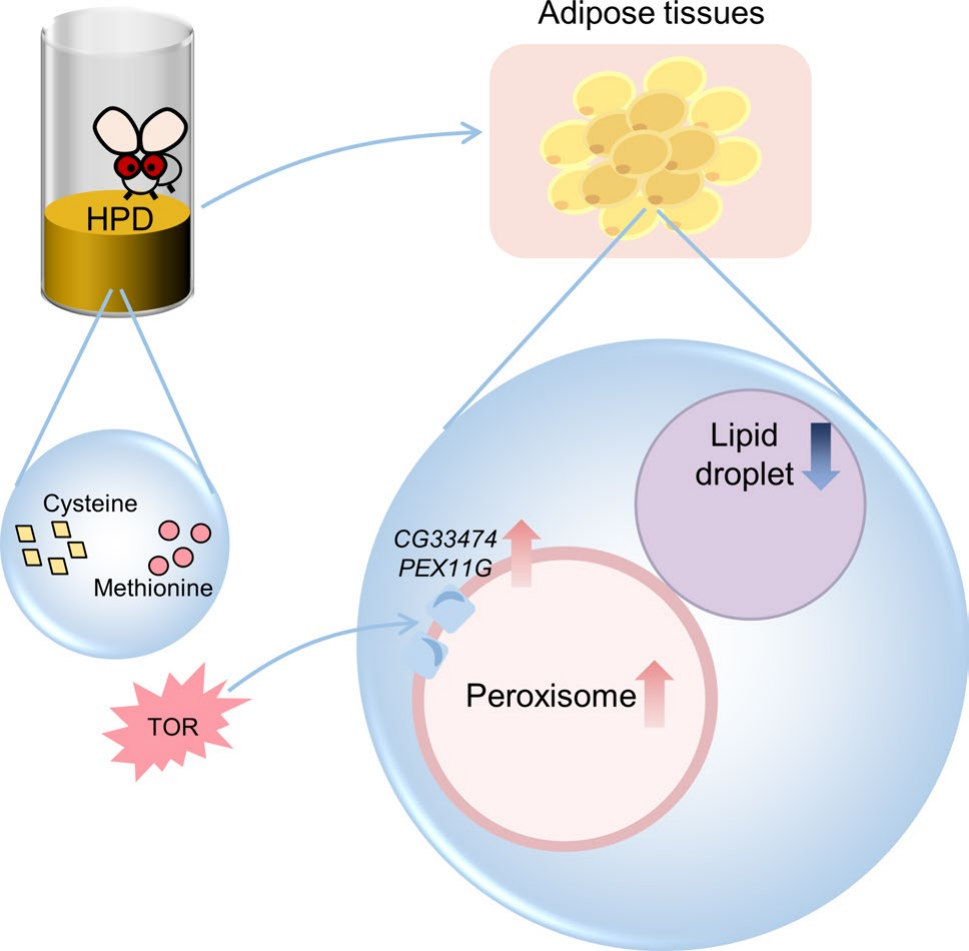
HPD, with cysteine and methionine serving as key amino acids, specifically elevates peroxisome levels in the adipose tissues of *Drosophila* by inducing *CG33474* expression. *CG33474*/*PEX11G* performs two essential biological roles in an evolutionarily conserved manner: firstly, overexpression of *CG33474*/*PEX11G* leads to increased peroxisome size; secondly, *CG33474*/*PEX11G* promotes the breakdown of LDs in a cell-autonomous manner (by strengthening peroxisome-LD interaction). Furthermore, TOR signaling is required for cysteine- and methionine-induced *CG33474*/*PEX11G* expression.

**Supplementary Information:**

The online version contains supplementary material available at 10.1007/s00018-024-05226-y.

## Introduction

Epidemiological studies have established correlations between obesity and a range of chronic diseases, including cardiovascular disease [1], multiple types of cancer [2], and diabetes mellitus [3]. Among various dietary strategies for weight management, high-protein diet (HPD) is frequently utilized since it is more likely to create satiety than carbohydrates and fats [4, 5]. Protein, one of the three primary macronutrients, is vital for maintaining body function. However, excessive protein intake may also pose detrimental effects on human health. For example, high protein ingestion can increase glomerular pressure and damage the glomerular structure, causing or aggravating chronic kidney diseases [6]. Additionally, increased protein intake can elevate blood and tissue amino acid levels, ultimately leading to atherosclerosis [7]. Besides, excessive protein consumption may induce negative outcomes (such as type-2 diabetes) by overstimulating the mTOR (mechanistic target of rapamycin) signaling pathway and concurrently inhibiting the FGF21 (fibroblast growth factor 21) signaling pathway [8]. Therefore, to fully evaluate the benefits and risks of HPD, comprehensive knowledge about its biological effects is imperative.

Peroxisomes perform several crucial functions in eukaryotic cells, including β-oxidation of very long-chain fatty acids, α-oxidation of branched-chain fatty acids, biosynthesis of ether phospholipids and bile acids, and elimination of reactive oxygen species [9]. Under normal circumstances, the half-life of peroxisomes is approximately 2 days [10], indicating that peroxisome biogenesis and degradation are highly dynamic processes. Peroxisomes can rapidly adapt to cell nutritional changes by altering their size, number, morphology, and protein composition [11]. The *PEX* genes encode diverse peroxin proteins, including matrix proteins, cytosolic receptors, and peroxisomal membrane proteins (PMPs) [12], thereby playing pivotal roles in maintaining the biogenesis and functions of peroxisomes. Several *PEX* genes, such as *PEX19* and *PEX5*, are regulated by nutrient status like lipids and fasting [13, 14]. Nonetheless, how high protein consumption or particular amino acids affect peroxisomal function and dynamics remains largely unexplored.

The organs and metabolic pathways involved in energy metabolism are similar between humans and *Drosophila melanogaster*, making *Drosophila* a helpful tool for elucidating the molecular mechanisms underlying nutrient sensing and regulation [15]. Using *Drosophila* as a model organism, we investigated the effect of HPD on peroxisomes. We found that HPD specifically elevates peroxisome levels in the adipose tissues and further identified cysteine and methionine as the primary amino acids responsible for this effect. Employing transcriptomics and genetic methods, we discovered that knocking down *CG33474*, a peroxisomal fission gene whose mammalian homology is *PEX11G*, suppresses the HPD-induced peroxisome elevation, indicating that the elevation of peroxisome levels triggered by HPD requires the presence of *CG33474*. *CG33474*/*PEX11G* exerts two pivotal biological functions in a preserved manner across species: firstly, *CG33474*/*PEX11G* overexpression results in increased peroxisome size; secondly, *CG33474*/*PEX11G* is required for cysteine- and methionine-induced fat loss, and overexpressing *CG33474*/*PEX11G* alone is sufficient to reduce lipid levels. Moreover, the intracellular nutrient sensor TOR signaling is required for cysteine- and methionine-induced *CG33474*/*PEX11G* expression.

In summary, this research established connections between HPD, peroxisome elevations, and fat loss, with *CG33474*/*PEX11G* identified as a key mediator. This study will not only enhance our understanding of the biological effects of HPD and the molecular mechanisms underlying the fat-reducing functions of specific amino acids but also provide potential targets for treating metabolic-related diseases.

## Materials and methods

### Fly husbandry and genetic manipulations

Flies were maintained at 25 ℃ and 50% humidity on a standard cornmeal food (the recipe for 1 L food is: 56 g cornmeal, 40 g yeast, 88 g glucose, 10 g agar, and 1.25 g nipagin) with a 12-h light/dark cycle. The *Gal4*/*UAS* (Upstream Activating Sequence) binary system [16] was applied to manipulate transgene expression in various tissues, with experimental crosses conducted at 25 ℃. FLP-out strains were crossed with indicated UAS-transgene strains at 25 ℃ to generate clones labeled with fluorescence. 5–7-day-old mated flies or 3rd instar larvae were used in all experiments, with detailed descriptions provided in the corresponding figure legends.

Fly strains utilized in this study: *w*^*1118*^ (lab stock), *lpp-Gal4* (lab stock), *hsFLP*; *AyGal4*, *UAS-GFP* (lab stock), *hsFLP*; *AyGal4*, *UAS-RFP* (lab stock), *UAS-CG33474-3*×*Flag* (this study), *CG33474-Gal4* (this study), *Ubi-GFP-PTS1* (this study), *UAS-GFP* (BDSC, #52262), *UAS-RFP* (BDSC, #8545), *UAS-Luciferase-RNAi* (BDSC, #31603), *UAS-lacZ* (lab stock), *UAS-CG33474-RNAi* (BDSC, #64672), *UAS-Rheb-RNAi* (BDSC, #33966), *UAS-Pex19-RNAi* (VDRC, #22064), *UAS-bmm-RNAi* (VDRC, #37877), *UAS-Sod1-RNAi* (BDSC, #34616), *UAS-Sod2-RNAi* (BDSC, #25969), *UAS-Rheb* (BDSC, #9688), and *UAS-GFP-PTS1* (BDSC, #64248).

### Dietary manipulations

Specific diets utilized in this study are described below.

The recipe for Normal Diet (ND, 100 mL): 1 g agar, 8 g brewer’s yeast, 2 g yeast extract (YE), 2 g peptone, and 5.1 g sucrose [17].

The recipe for High-protein Diet (HPD, 100 mL): 1 g agar, 8 g brewer’s yeast, 2 g yeast extract, 2 g peptone, 5.1 g sucrose, 6.6 g Crisco, and 15 g soy protein (GNC) [17].

The difference in protein content between HPD and ND (per 100 mL) lies in the inclusion of 15 g soy protein, which equates to 13 g protein.

Protein content (30% YE, 100 mL): 100 × 30% × 40.7% = 12.21 g.

### Cell culture and transfection

*Drosophila* S2 cells (lab stock) were cultured in Schneider medium in a humidified incubator at 25 ℃. Schneider medium was prepared by supplementing Schneider’s Insect Medium (Sigma Aldrich) with 10% fetal bovine serum (FBS, Biological Industries), 1% GlutaMAX (Gibco), and 1% penicillin/streptomycin (Gibco).

HEK293T cells (lab stock) and U2OS cells (lab stock) were cultured in high-glucose Dulbecco’s modified Eagle medium (DMEM, Gibco) supplemented with 10% FBS, 1% GlutaMAX, and 1% penicillin/streptomycin in a humidified incubator at 37 ℃ with 5% CO_2_.

Effectene transfection reagent (QIAGEN) and jetPRIME DNA transfection reagent (Polyplus) were utilized for plasmid transfection into *Drosophila* S2 cells and mammalian cells, respectively, for a duration of 24 h. To assess lipid droplets (LDs) in mammalian cells, cells were transfected with plasmids and treated with oleic acid (OA, Sigma)-containing medium for 24 h.

### Molecular cloning

For the generation of the *UAS-CG33474-3*×*Flag* transgenic line, *CG33474* coding sequence was acquired from the *Drosophila* complementary DNA (cDNA) (reverse transcribed from total RNA extracted from *w*^*1118*^ flies) using polymerase chain reaction (PCR). The *CG33474* coding sequence was cloned into the IVS-p10 vector (lab stock) digested with XbaI (Takara). The 3× Flag sequence (DYKDHDGDYKDHDIDYKDDDDK) was inserted into the C-terminus of the recombinant product. This tagged construct was further transferred to the pUAST-attB Gateway destination vector (lab stock) using the LR Clonase Enzyme Mix (Invitrogen) according to the manual. Ultimately, the expression construct was injected into the embryos of *y*^*1*^*w*^*67c23*^; *P(CaryP)attP2* (BDSC, #8622) for phiC31-mediated recombination at an *attP* insertion site on the third chromosome by UniHuaii Corporation.

For the generation of the *Ubi-GFP-PTS1* transgenic line, GFP coding sequence (lab stock) was cloned into the IVS-p10 vector digested with XbaI. The PTS1 sequence (PEALIKSMTSKL) was inserted into the C-terminus of the recombinant product. This tagged construct was further transferred to the pUbi-attB Gateway destination vector (lab stock) using the LR Clonase Enzyme Mix. Ultimately, the expression construct was injected into the embryos of *PBac(attP-9A)VK00027* (BDSC, #9744) for phiC31-mediated recombination at an *attP* insertion site on the third chromosome by UniHuaii Corporation.

*UAS-CG33474-3*×*Flag* and *Ubi-GFP-PTS1* offsprings were screened for red eyes (selection marker mini-white).

A CRISPR-mediated knock-in strategy was used to generate the *CG33474-Gal4* knock-in line. Briefly, three single guide RNAs (sgRNAs) targeting the start coding region of *CG33474* were designed using an online tool (https://www.flyrnai.org/crispr3/web/). Three pairs of oligonucleotides that contained the sgRNA sequence were individually introduced into the pCFD3 vector (lab stock) digested with BbsI (Thermo Fisher Scientific). Two 1-kb-long homology arms were amplified from the *Drosophila* genome (genomic DNA extracted from *w*^*1118*^ flies) and cloned into the pDonor-T2A-Gal4 vector (lab stock) to construct the donor plasmid. Subsequently, three sgRNA-bearing vectors and the donor plasmid were co-injected into the embryos of *vas-Cas9* flies (BDSC, #55821) by UniHuaii Corporation. F_0_ flies were crossed with the balancer line (*If*/*CyO*; *MKRS.Sb*/*TM6B.Tb*), and the F_1_ offspring were screened using genomic PCR with oligos targeting the Gal4 region.

For the design of PEX11G-GFP plasmid, *PEX11G* coding sequence was PCR-amplified from the pENTR223-PEX11G template (purchased from Miaoling Biology) and then cloned into the pcDNA3.1 vector (lab stock). Subsequently, GFP coding sequence was inserted into the C-terminus of the recombinant product.

All the DNA constructions were confirmed by sequencing at Sangon Biotech.

### RNA-Seq

Three independent biological replicates were prepared for each experimental group. RNA-Seq analysis was conducted by GENEWIZ Corporation. Briefly, total RNA was acquired from ten adult flies using TRIzol Reagent (Invitrogen) following the manufacturer’s instruction. The quality and integrity of RNA were evaluated using a NanoDrop 2000 Spectrophotometer (Thermo Fisher Scientific) and the RNA 6000 Pico Kit (Agilent). Libraries were prepared using the TruSeq Stranded mRNA Kit (Illumina) according to the manual. These libraries were then loaded on a NovaSeq 6000 System (Illumina) for sequencing using a 150 bp paired-end configuration. The RNA-Seq data were analyzed using the DESeq2 with default settings [18]. Genes were considered differentially expressed if they exhibited a *p* value <0.05 and a fold change >2.

### RT-qPCR

Total RNA was extracted using TRIzol Reagent and subsequently quantified with a NanoDrop 2000 Spectrophotometer. cDNA was synthesized from 500 ng of total RNA using the HiScript II 1st Strand cDNA Synthesis Kit (+gDNA wiper) (Vazyme) following the instructions. RT-qPCR was performed using the AceQ qPCR SYBR Green Master Mix (Low ROX Premixed) (Vazyme) on a QuantStudio 3 System (Thermo Fisher Scientific). Relative mRNA expression levels were calculated using the comparative C_T_ method [19] and normalized to *RpL23* (*Drosophila*) or *Beta-actin* (HEK293T) transcript levels. In all cases, three independent biological replicates were performed. Primer sequences utilized in RT-qPCR are listed in Table S1 and Table S2.

### Immunostainings

For immunostaining of *Drosophila* tissues, dissections were performed in cold phosphate-buffered saline (PBS, Biosharp). For immunostaining of tissue sections, specimens were embedded in OCT (Sakura Rinetek UAS) and snap-frozen. Once hardened, samples were sliced into 10 μm sections using a freezing cryostat (Leica CM1950). For immunostaining of cells, cells were initially washed with PBS. Thereafter, all samples were fixed with 4% paraformaldehyde (PFA, Sango Biotech) for 20 min at room temperature. After fixation, samples were washed three times with PBST (0.5% Triton X-100 in PBS, 5 min each), then blocked with 2% bovine serum albumin (BSA, Sango Biotech) in PBST for 1 h at room temperature. Samples were subsequently incubated with primary antibodies diluted in PBST containing 2% BSA at 4 ℃ overnight. Following incubation, samples were washed three times with PBST (5 min each), then incubated with appropriate Alexa Fluor-conjugated secondary antibodies and DAPI (1 μg/mL; Invitrogen) diluted in PBST containing 2% BSA for 1 h at room temperature followed by the same washing procedures above. For staining of LDs and filamentous actin (F-actin), samples were stained with Nile Red (10 μg/mL; Invitrogen), LipidTOX (1:1000; Invitrogen), or Alexa Fluor 488 Phalloidin (1:500; Cell Signaling Technology) for 30 min at room temperature followed by the same washing procedures above. Samples were then mounted on slides using ProLong Gold antifade reagent (Thermo Fisher Scientific) and imaged under the Leica DMi8 microscope.

Antibodies were used at the following dilutions: mouse anti-Flag (1:500; Proteintech), chicken anti-GFP (1:500; Aves Labs), rabbit anti-PMP70 (1:500; for staining mammalian cells; Invitrogen), rabbit anti-Pmp70 (1:200; for staining *Drosophila* tissues; this study; two rabbits were immunized with one peptide of *Drosophila* Pmp70 (DGRGSYEFATIDQDKDHFGS), and the antisera were affinity purified by Diaan Corporation.), rabbit anti-Catalase (1:500; Proteintech), rabbit anti-PEX5 (1:500; Proteintech), rabbit anti-PEX19 (1:500; Proteintech), anti-chicken Alexa Fluor 488 (1:1000; Invitrogen), anti-mouse Alexa Fluor 488 (1:1000; Invitrogen), anti-mouse Alexa Fluor 555 (1:1000; Invitrogen), anti-rabbit Alexa Fluor 555 (1:1000; Invitrogen), anti-mouse Alexa Fluor 647 (1:1000; Invitrogen).

### DHE stainings

The Dihydroethidium (DHE) dye (Invitrogen) was used to assess ROS levels. Samples were incubated with 5 μM DHE for 60 min at 37 ℃, washed three times with PBS, and imaged immediately using the Leica DMi8 microscope.

### Detection of lipid peroxidation

The C11 BODIPY 581/591 probe (Invitrogen) was employed to evaluate lipid peroxidation. Tissues were incubated with 10 μM C11 BODIPY 581/591 for 60 min at room temperature, washed three times with PBS, and promptly imaged using the Leica DMi8 microscope.

### Western blot

Samples were homogenized in RIPA Lysis Buffer (Biosharp) supplemented with protease inhibitor cocktail (TargetMol) and phosphatase inhibitor cocktail (TargetMol) and incubated on ice for 30 min. After a 12,000 g centrifugation for 15 min, the supernatants were collected as total proteins. Protein concentrations were determined using the BCA Protein Quantification Kit (Vazyme). Proteins were mixed with 5× Sample Loading Buffer (Sangon Biotech) and heated at 95 ℃ for 5 min. Equal amounts of proteins post-boiling were loaded into 10% sodium dodecyl sulfate–polyacrylamide gel electrophoresis (SDS-PAGE) and blotted into polyvinylidene difluoride (PVDF) membranes (Merck Millipore). The PVDF membranes were then blocked in TBST (0.5% Tween-20 in TBS) containing 5% Non-Fat Powdered Milk (Sango Biotech) for 1 h at room temperature. Following blocking, the PVDF membranes were incubated with primary antibodies diluted in TBST containing 5% Non-Fat Powdered Milk at 4 ℃ overnight. After incubation, the PVDF membranes were washed three times with TBST (5 min each), then incubated with appropriate horseradish peroxidase (HRP)-conjugated secondary antibodies diluted in TBST containing 5% Non-Fat Powdered Milk for 1 h at room temperature followed by the same washing steps previously. The PVDF membranes were then visualized using the SuperSignal West Pico PLUS Chemiluminescent Substrate (Thermo Fisher Scientific) and detected by the Tanon 5200 Chemiluminescent Imaging System. Expression of tubulin was utilized as a loading control.

Antibodies were used at the following dilutions: rabbit alpha-tubulin (1:5000; Proteintech), rabbit phosphor-4E-BP1 (Thr37/46) (1:1000, Cell Signaling Technology), rabbit Non-phospho-4E-BP1 (Thr46) (1:1000, Cell Signaling Technology), rabbit DRP1 (C-terminal) (1:5000; Proteintech), rabbit FIS1 (1:5000; Proteintech), rabbit GFP (1:2000; Proteintech), rabbit PMP70 (1:5000; Invitrogen), rabbit PEX5 (1:1000; Proteintech), rabbit PEX19 (1:1000; Invitrogen), rabbit ACOX1 (1:5000; Proteintech), rabbit Sod1 (1:5000; Proteintech), rabbit Catalase (1:5000; Proteintech), and HRP-conjugated goat anti-rabbit (1:5000; Invitrogen).

### Ex vivo experiments

Fat bodies from 3rd instar wandering larvae were dissected in Schneider medium and transferred to wells containing fresh Schneider medium in a humidified incubator at 25 ℃. For drug treatment, compounds were added to Schneider medium, filtered through 0.22 μm needle filters, and immediately utilized. After incubation, fat bodies were stained with DAPI (1 μg/mL in PBS) for 10 min, mounted on slides using ProLong Gold antifade reagent, and photographed on the Leica DMi8 microscope.

### Chemical reagents

L-Alanine (CAS No. 56-41-7), L-Arginine (CAS No. 74-79-3), L-Asparagine (CAS No. 70-47-3), L-Aspartic acid (CAS No. 56-84-8), L-Cysteine (CAS No. 52-90-4), L-Glutamic acid (CAS No. 56-86-0), L-Glutamine (CAS No. 56-85-9), L-Histidine (CAS No. 71-00-1), L-Isoleucine (CAS No. 73-32-5), L-Leucine (CAS No. 61-90-5), L-Lysine (CAS No. 56-87-1), L-Methionine (CAS No. 63-68-3), L-Phenylalanine (CAS No. 63-91-2), L-Proline (CAS No. 147-87-3), L-Serine (CAS No. 56-45-1), L-Threonine (CAS No. 72-19-5), L-Tryptophan (CAS No. 73-22-3), L-Tyrosine (CAS No. 60-18-4), L-Valine (CAS No. 72-18-4), and Glycine (CAS No. 56-40-6) were purchased from MACKLIN. MHY1485 (CAS No. 326914-06-1) and Rapamycin (CAS No. 53123-88-9) were purchased from APExBIO.

### TAG assay

Ten larvae or flies (one sample) were homogenized in 150 μL cold isopropanol. The homogenized samples were then centrifuged at 12,000 g for 10 min at 4 ℃. Next, triglyceride (TAG) levels were assayed using the Triglyceride (TG) Colorimetric Assay Kit (Elabscience) according to the manual. Protein contents were determined by the BCA Protein Quantification Kit for normalization.

### Quantifications of peroxisomes and LDs

Quantifications of peroxisomes and LDs were performed using Fiji (NIH), with the data normalized to control groups. Procedures are described below: (1) File→open; (2) Image→type→8-bit; (3) Image→adjust→threshold; (4) Select the region of interest (ROI); (5) Analyze→analyze particles. Ensure that the boxes marked “Display results”, “Clear results”, “Summarize” and “Add to Manager” are ticked.

### Statistical analyses

Data were analyzed using Origin (OriginLab), GraphPad Prism (GraphPad Software), and Excel (Microsoft). Statistical significance was assessed using two-tailed Student’s *t* test, Fisher’s Exact Test, or one-way analysis of variance (ANOVA), and was indicated with * (* for *p* < 0.05, ** for *p* < 0.01, *** for *p* < 0.001, and **** for *p* < 0.0001). Values are presented as mean ± SEM.

## Results

### Cysteine and methionine are key factors in HPD-induced peroxisome elevation

**Fig. 1:**
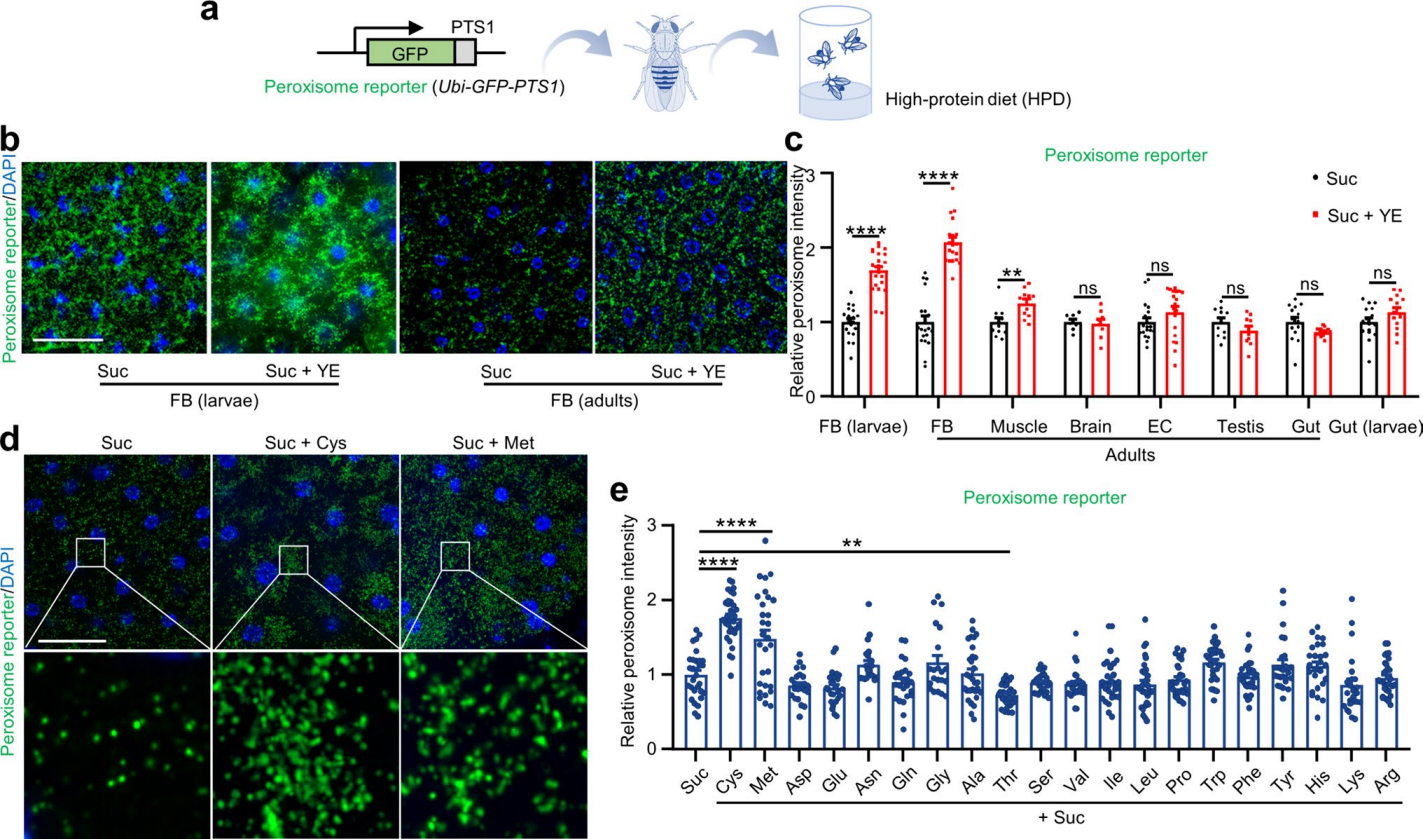
Cysteine and methionine are key factors in HPD-induced peroxisome elevation. **a** Scheme of the construction of the *Ubi-GFP-PTS1* line and the experimental setup. **b** Peroxisome levels in the adipose tissues of *Ubi-GFP-PTS1* 3rd instar larvae and female flies fed with Suc (5% sucrose) or Suc + YE (5% sucrose + 30% yeast extract) for 36 h. DAPI (blue) labeled nuclei. Scale bar: 100 μm. **c** Relative fluorescence intensities of peroxisomes in **b** and Fig. S2. From left to right: 19, 21, 19, 20, 11, 11, 7, 8, 19, 19, 11, 10, 14, 12, 16, and 14 views. **d** Peroxisome levels in the adipose tissues of *Ubi-GFP-PTS1* female flies 36 h following the designated treatments. The boxed areas were enlarged to the lower panel. DAPI (blue) labeled nuclei. Suc: 5% sucrose; Suc + Cys: 5% sucrose + 25 mM cysteine; Suc + Met: 5% sucrose + 25 mM methionine. Scale bar: 100 μm. **e** Relative fluorescence intensities of peroxisomes in Fig. S3. From left to right: 30, 33, 29, 24, 26, 22, 25, 21, 30, 32, 27, 28, 31, 32, 32, 32, 32, 27, 26, 25, and 31 views. Two-tailed Student’ s *t* test (**c**) or one-way ANOVA (**e**) were performed. ** *p* < 0.01; **** *p* < 0.0001; ns, not significant

To explore the impacts of high protein intake on peroxisomes across various *Drosophila* tissues, we generated a transgenic peroxisomal reporter line, *Ubi-GFP-PTS1*, that ubiquitously labels peroxisomes in all fly tissues (Fig. 1a). *Ubi-GFP-PTS1* adult flies and 3rd instar larvae were provided with either a normal diet (ND) or a high-protein diet (HPD) formulated by Thomas J Baranski et al. [17]. 36 h later, major fly organs were dissected followed by the measurement of peroxisome levels. Compared to the control diet, HPD led to an increased intensity of green fluorescent protein (GFP) signal within the adipose tissues of *Ubi-GFP-PTS1* larvae and adult flies (Fig. S1a, b, i), indicating an increase in peroxisome levels under HPD feeding. No significant differences in peroxisome levels were observed in the adult muscles, adult brains, adult egg chambers, adult testes, and larval guts of *Ubi-GFP-PTS1* line fed with HPD compared to the control diet (Fig. S1c–f, h, i). However, a slight decrease in peroxisome levels was observed in the adult guts of *Ubi-GFP-PTS1* strain when subjected to HPD (Fig. S1g, i).

Yeast extract (YE) serves as the major protein source for *Drosophila* in natural and experimental environments [20]. To further confirm the effects of HPD on peroxisomes, we adopted a minimal food medium containing 5% sucrose and adjusted the YE content to 30% (based on the calculations derived from ND and HPD; see methods). Subsequently, *Ubi-GFP-PTS1* adult flies and 3rd instar larvae were fed with or without 30% YE for 36 h before examination. Consistent with our previous findings, YE feeding promoted peroxisome elevation in the adipose tissues of *Ubi-GFP-PTS1* larvae and adult flies compared to the 5% sucrose control diet (Fig. 1b, c). Conversely, no significant changes in peroxisome levels were observed in adult brains, adult egg chambers, adult testes, adult guts, and larval guts in this YE-fed *Ubi-GFP-PTS1* line compared to the 5% sucrose control diet, whereas adult muscles showed a slight increase in peroxisome levels (Fig. S2; Fig. 1c). Collectively, HPD specifically increases peroxisome levels in the adipose tissues of *Drosophila*.

Theoretically, the upregulation of peroxisome levels induced by HPD could be attributed to the overall increase in amino acid concentrations. However, there is a possibility that certain amino acids play a key role in promoting peroxisome elevation. To test this hypothesis, we fed *Ubi-GFP-PTS1* female flies with 20 individual amino acids for a duration of 36 h and measured peroxisome levels in their adipose tissues. Among the 20 naturally occurring L-amino acids tested, only cysteine and methionine significantly elevated peroxisome levels in the adipose tissues, whereas threonine slightly lowered peroxisome levels within the adipose tissues (Fig. 1d, e; Fig. S3), indicating that cysteine and methionine are key factors in HPD-induced peroxisome elevation.

### HPD elevates peroxisome levels by triggering *CG33474* expression

**Fig. 2:**
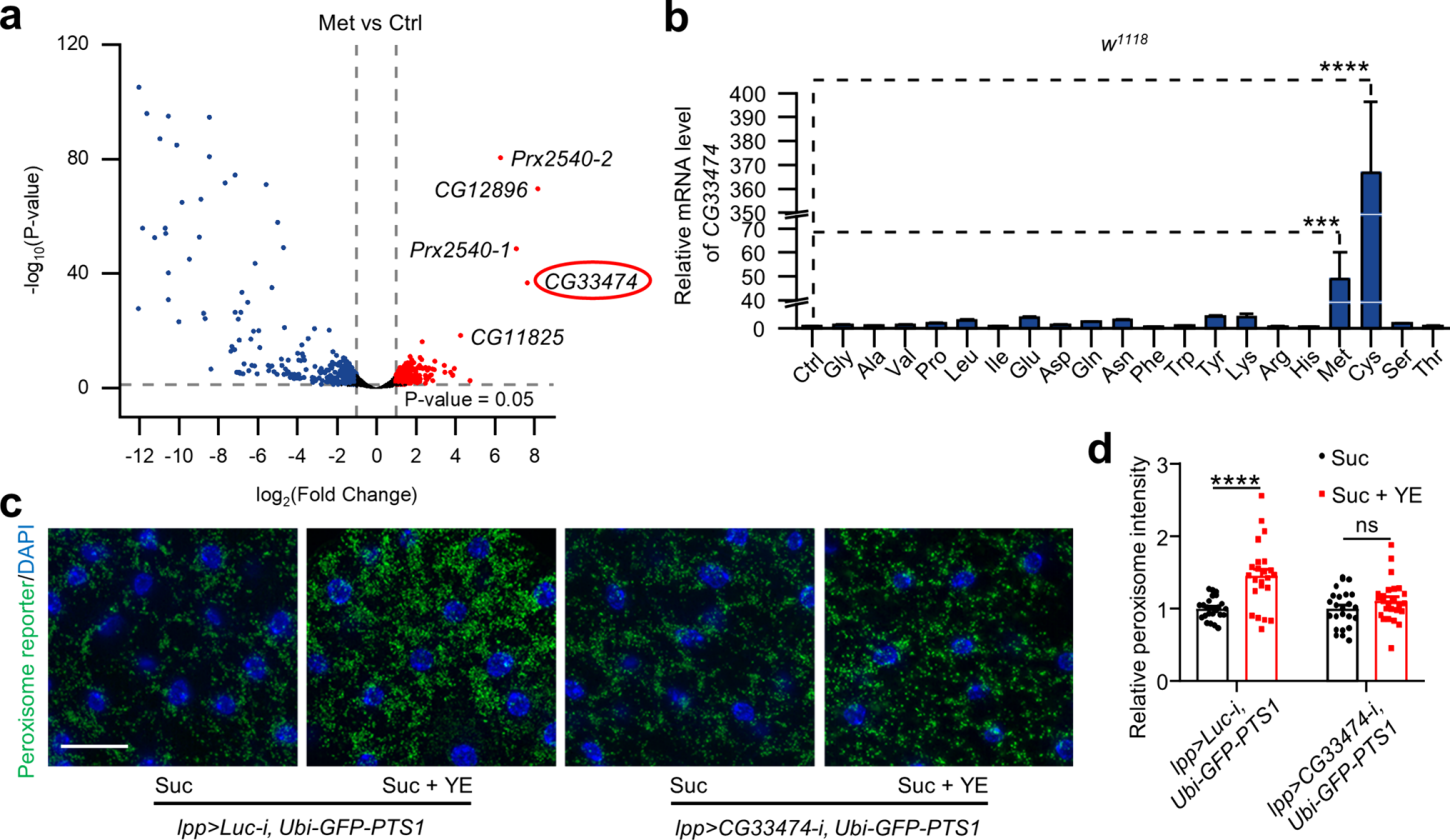
HPD elevates peroxisome levels by triggering *CG33474* expression. **a** Volcano plot depicting the differentially expressed genes in *w*^*1118*^ female flies treated with Ctrl (1% agarose) or Met (1% agarose + 25 mM methionine) for 36 h. The red and blue dots represented significantly upregulated and downregulated genes under methionine treatment, respectively. **b** Relative *CG33474* mRNA levels in *w*^*1118*^ female flies fed with Ctrl (1% agarose) or various amino acids (1% agarose + 25 mM individual amino acids) for 36 h. *n* = 3. **c** Peroxisome levels in the adipose tissue of the designated genotypes fed with Suc (5% sucrose) or Suc + YE (5% sucrose + 30% yeast extract) for 36 h. DAPI (blue) labeled nuclei. Scale bar: 100 μm. **d** Relative fluorescence intensities of peroxisomes in **c**. From left to right: 25, 23, 24, and 25 views. One-way ANOVA (**b**) or two-tailed Student’s *t* test (**d**) were performed. *** *p* < 0.001; **** *p* < 0.0001; ns, not significant

The results above suggested that methionine and cysteine might increase peroxisome levels by activating specific *Pex* genes. To identify potential peroxisomal genes responsible for increasing peroxisome levels under HPD feeding, we selected methionine, the more extensively studied amino acid [21], and conducted RNA-Seq analyses on *w*^*1118*^ female flies that were fed with or without methionine for a period of 36 h. Compared to the methionine-absent group, over 700 genes were significantly changed in the methionine-fed group. Analyses of the differentially expressed genes revealed that the transcript level of *CG33474*, a peroxisomal fission gene, was significantly increased in flies fed a methionine-rich diet (Fig. 2a). We further verified the RNA-Seq results by RT-qPCR. Consistent with the RNA-Seq data, RT-qPCR results demonstrated that cysteine and methionine, but not other amino acids, effectively induced the expression of *CG33474* (Fig. 2b).

Next, we quantified the transcript levels of four distinct categories of peroxisome-associated genes in *w*^*1118*^ female flies following a-36-h cysteine or methionine diet: peroxisomal fission genes (*Fis*, *Pex11ab*, and *Pex11c*), peroxisomal organization-related genes (*ABCD*, *Pmp70*, *CG31454*, *CG31259*, and *CG11737*), genes involved in peroxisomal membrane assembly (*Pex3*, *Pex16*, and *Pex19*), and genes responsible for peroxisomal protein import (*Pex1*, *Pex2*, *Pex5*, *Pex6*, *Pex7*, *Pex10*, *Pex12*, *Pex13*, and *Pex14*), respectively [22]. None of these genes were concurrently induced by both methionine and cysteine, unlike *CG33474* (Fig. S4). Together, it was reasonable to infer that *CG33474* might be a crucial peroxisomal gene in HPD-induced peroxisome elevation.

To ascertain whether HPD-induced peroxisome elevation depends on *CG33474*, we knocked down *Luciferase* or *CG33474* specifically in the adipose tissues and subsequently fed female flies of these genotypes with or without 30% YE for 36 h. Compared to the control diet, knocking down *Luciferase* in the fat body (*lpp-Gal4>UAS-Luciferase-RNAi*, *Ubi-GFP-PTS1*) led to a significant increase in peroxisome levels under HPD feeding as expected (Fig. 2c, d). On the contrary, knockdown of *CG33474* in the fat body (*lpp-Gal4>UAS-CG33474-RNAi*, *Ubi-GFP-PTS1*) failed to elevate peroxisome levels under HPD feeding (Fig. 2c, d), suggesting that HPD-induced peroxisome elevation requires *CG33474*.

### Cysteine and methionine are key factors in HPD-induced *CG33474* expression

**Fig. 3:**
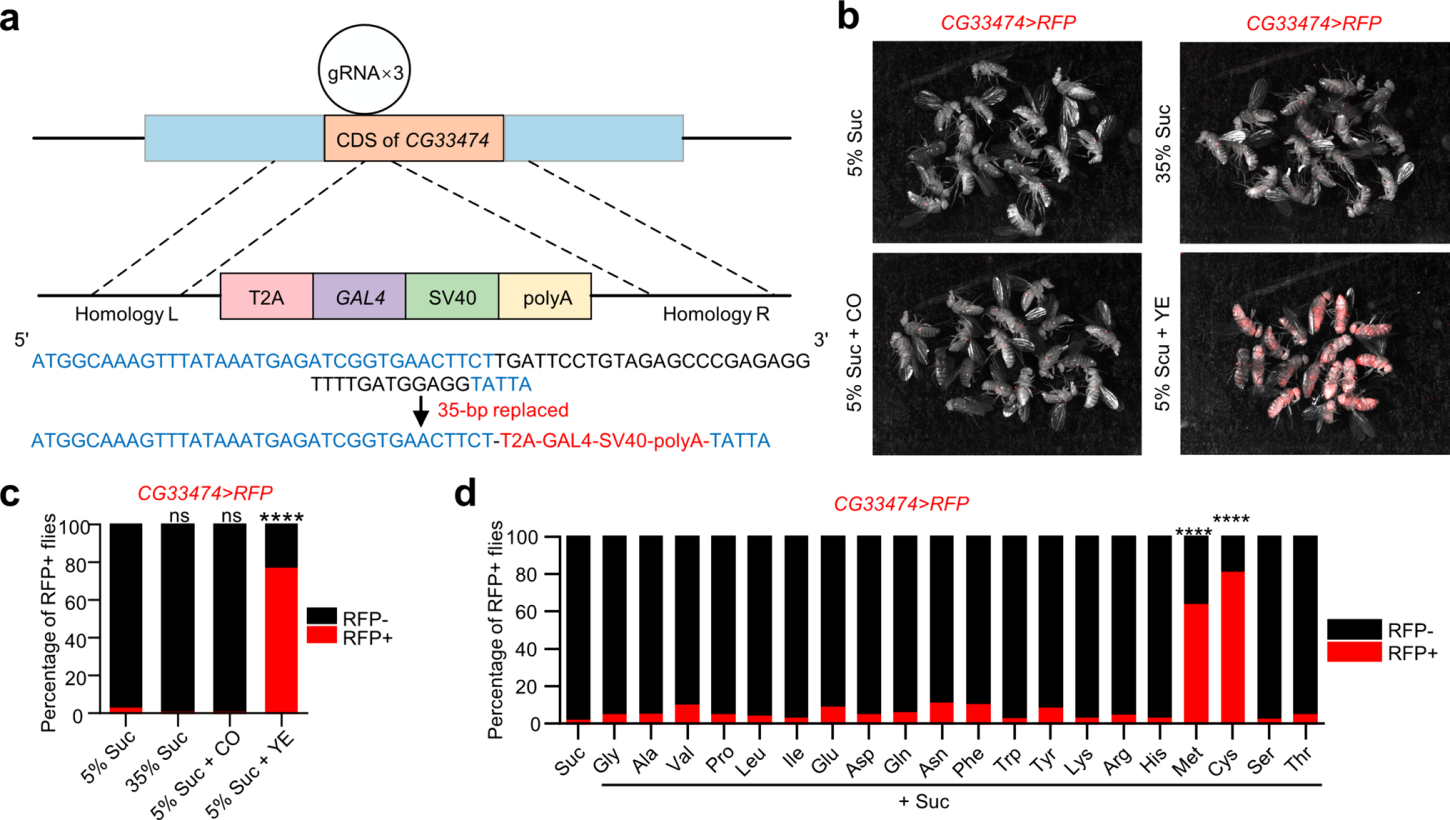
Cysteine and methionine are key factors in HPD-induced *CG33474* expression. **a** A diagram represents the *CG33474-Gal4* knock-in approach. Represent images (**b**) and quantification (**c**, from left to right: 85, 84, 82, and 81 flies) of *CG33474-Gal4>UAS-RFP* female flies expressing RFP 36 h following the indicated treatments. 5% Suc: 5% sucrose; 35% Suc: 35% sucrose; 5% Suc + CO: 5% sucrose + 15% coconut oil; 5% Suc + YE: 5% sucrose + 30% yeast extract. **d** Percentage of *CG33474-Gal4>UAS-RFP* female flies expressing RFP 36 h following Suc (5% sucrose) or Suc + amino acids (5% sucrose + 25 mM individual amino acids) diets. From left to right: 68, 54, 68, 56, 52, 60, 51, 51, 53, 61, 51, 55, 54, 43, 51, 56, 51, 85, 77, 60, and 52 flies. Fisher’s Exact Test was performed. **** *p* < 0.0001; ns, not significant

To better visualize the expression of *CG33474* in vivo, we generated a *CG33474-Gal4* knock-in (KI) line using the CRISPR-Cas9 system [23] (Fig. 3a). Notably, the *CG33474-Gal4* line also functions as a *CG33474* mutant line because the coding region of *CG33474* was destroyed (Fig. 3a). We verified the mutation effect of this line through RT-qPCR (Fig. S5a). Additionally, the *CG33474-Gal4* homozygous flies are viable and fertile, indicating that *CG33474* may not play essential roles under normal developmental conditions. *CG33474-Gal4* flies were crossed with *UAS-RFP* flies, and the offspring were used as a CG33474 fluorescent reporter line. Compared to ND feeding, *CG33474-Gal4>UAS-RFP* female flies under HPD revealed a significant increase in the percentage of RFP+ flies (flies displaying red fluorescence) (Fig. S5b, c), validating the efficacy of this CG33474 fluorescent reporter system. Next, we fed *CG33474-Gal4>UAS-RFP* female flies with various diets for 36 h. A significant increase in the proportion of RFP+ flies was observed under the YE condition (Fig. 3b, c), verifying that HPD triggers the expression of *CG33474*. However, *CG33474* exhibited no activation under high-sugar or high-fat diets, as shown by no increase in the percentage of RFP+ flies (Fig. 3b, c), indicating that the trigger of *CG33474* is HPD-specific. Next, we tested the roles of individual amino acids in inducing *CG33474* expression using *CG33474-Gal4>UAS-RFP* female flies. Consistent with previous results, dietary cysteine and methionine both significantly potentiated *CG33474* expression to a distinctly greater extent than any other amino acids (Fig. 3d), indicating that cysteine and methionine are key factors in HPD-induced *CG33474* expression.

Given that cysteine functions as a scavenger of reactive oxygen species (ROS) [24] and methionine serves both as a metabolic precursor of cysteine and a direct target of ROS [25], we investigated whether alterations in ROS levels could trigger *CG33474* expression. To this end, we fed *CG33474-Gal4>UAS-RFP* female flies with different reductive or oxidative compounds for 36 h. Neither the inhibition of ROS through the administration of Trolox or Vitamin C [26] nor the induction of ROS via Paraquat or H_2_O_2_ [27] was able to promote *CG33474* expression (Fig. S5d). Altogether, these data suggested that *CG33474* expression is probably triggered by certain metabolic products of cysteine and methionine, rather than changes in ROS levels.

Next, we explored whether methionine- and cysteine-induced *CG33474* expression is gender-specific. For this purpose, we fed *CG33474-Gal4>UAS-RFP* female and male flies with or without methionine or cysteine for 36 h. The results showed that in both sexes, the proportion of RFP+ flies significantly rose upon exposure to methionine or cysteine (Fig. S5e), indicating that cysteine- and methionine-induced *CG33474* expression is sexual-independent.

### CG33474/PEX11G proteins lead to increased peroxisome size

**Fig. 4:**
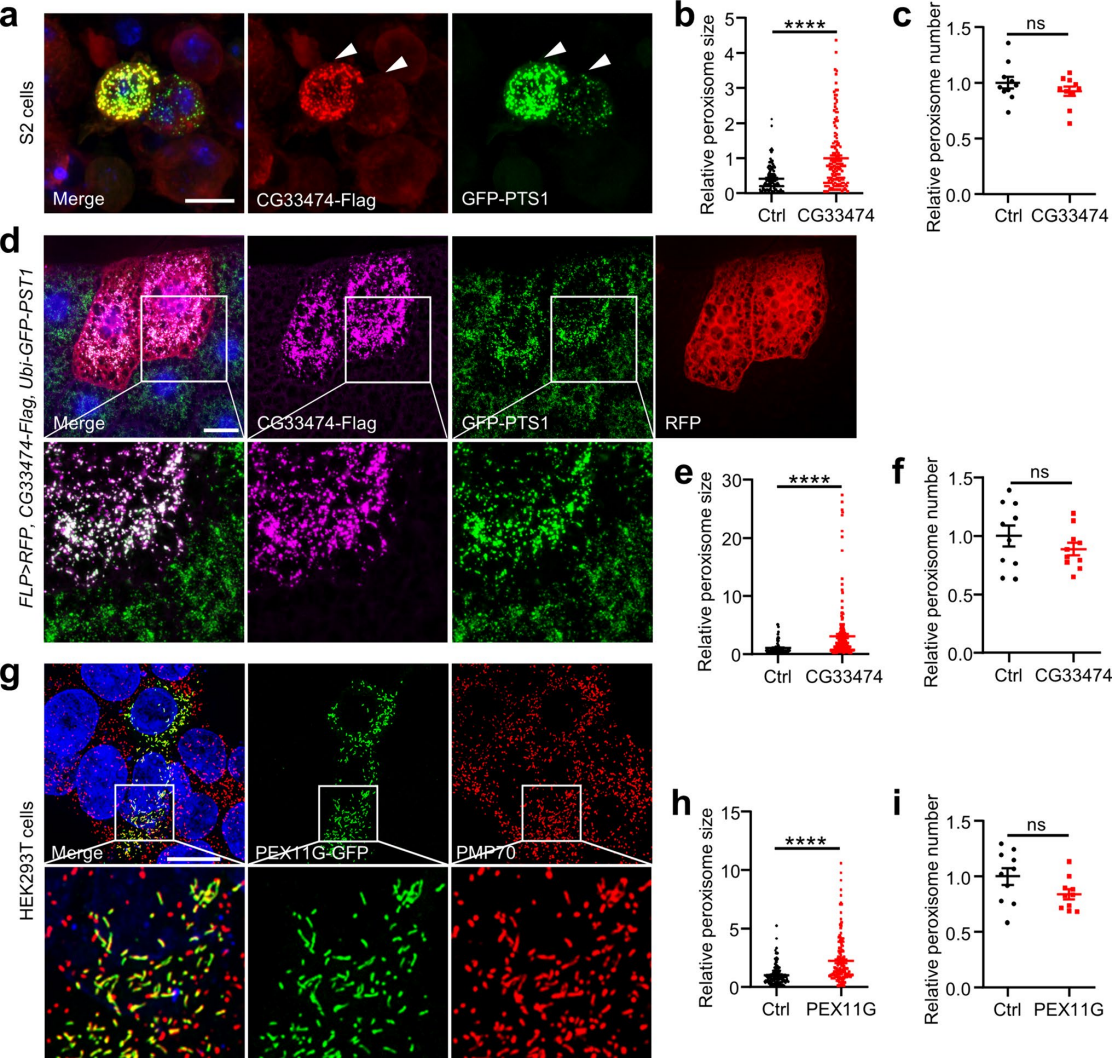
CG33474/PEX11G proteins lead to increased peroxisome size. **a** Fluorescence microscopy images of *Drosophila* S2 cells co-transfected with CG33474-Flag (red) and GFP-PTS1 encoding plasmids. DAPI (blue) labeled nuclei. GFP-PTS1 (green) indicated peroxisomes. Arrows indicated a significant difference between control and *CG33474-*expressing cells. Scale bar: 10 μm. Relative peroxisome size (**b**, *n* = 10 cells.) and number (**c**, *n* = 10 cells.) between control (Ctrl) and *CG33474* expressing cells (CG33474) in **a**. **d** Fluorescence microscopy images of fat bodies from 3rd instar larvae of the designated genotype that expressed CG33474-Flag (magenta). The boxed areas were enlarged to the lower panel. DAPI (blue) labeled nuclei. Clones were labeled by RFP (red). GFP-PTS1 (green) indicated peroxisomes. Scale bar: 20 μm. Relative peroxisome size (**e**, *n* = 10 cells.) and number (**f**, *n* = 10 cells.) between control (Ctrl) and *CG33474-*expressing cells (CG33474) in **d**. **g** Fluorescence microscopy images of HEK293T cells transfected with PEX11G-GFP encoding plasmid. The boxed areas were enlarged to the lower panel. DAPI (blue) labeled nuclei. PMP70 (red) indicated peroxisomes. Scale bar: 20 μm. Relative peroxisome size (**h**, *n* = 10 cells.) and number (**i**, *n* = 10 cells.) between control (Ctrl) and *PEX11G* expressing cells (PEX11G) in **g**. Two-tailed Student’s *t* test was performed. **** *p* < 0.0001; ns, not significant

To examine whether *CG33474* regulates the size of peroxisomes, we co-transfected CG33474-Flag and GFP-PTS1 (GFP fused to the PTS1 labeled peroxisomes) encoding plasmids into *Drosophila* S2 cells. The results demonstrated that CG33474 proteins localized to peroxisomes within S2 cells (Fig. 4a). Furthermore, *CG33474* overexpression resulted in increased peroxisome size in S2 cells (Fig. 4b), aligning with the previously documented juxtaposed elongated peroxisomes (JEPs) induced by PEX11G in mammalian cells [28].

To investigate whether *CG33474* modulates peroxisome size in vivo, we employed the FLP-out system to co-express CG33474-Flag and GFP-PTS1. Subsequently, we analyzed the impact of *CG33474* on peroxisomes using 3rd instar larvae of this specific genotype. The endogenous CG33474-Flag fusion proteins colocalized with GFP-PTS1, a marker of peroxisomes (Fig. 4d). Furthermore, adipocytes expressing *CG33474* also exhibited enlarged peroxisome size (Fig. 4e).

To further investigate whether the role of CG33474/PEX11G proteins in modifying peroxisome size is conserved across species, we transfected mammalian HEK293T cells with plasmids encoding CG33474-Flag and PEX11G-GFP, respectively. The results demonstrated that both *Drosophila* CG33474 (Fig. S6a) and mammalian PEX11G proteins (Fig. 4g) co-localized with the peroxisomal marker PMP70. Moreover, overexpression of either CG33474 (Fig. S6b) or PEX11G proteins (Fig. 4h) in HEK293T cells increased peroxisome size. Notably, overexpression of *CG33474* (Fig. 4c, f) or *PEX11G* (Fig. 4i) did not alter the number of peroxisomes in the respective model organism. However, a slight decrease in the number of peroxisomes was observed upon *CG33474* overexpression in HEK293T cells (Fig. S6c). Taken together, our findings proved that the cellular function of CG33474 and PEX11G proteins in altering peroxisome size is evolutionarily conserved between *Drosophila* and mammals.

### Cysteine- and methionine-induced *CG33474* expression is primarily in the adipose tissues

**Fig. 5:**
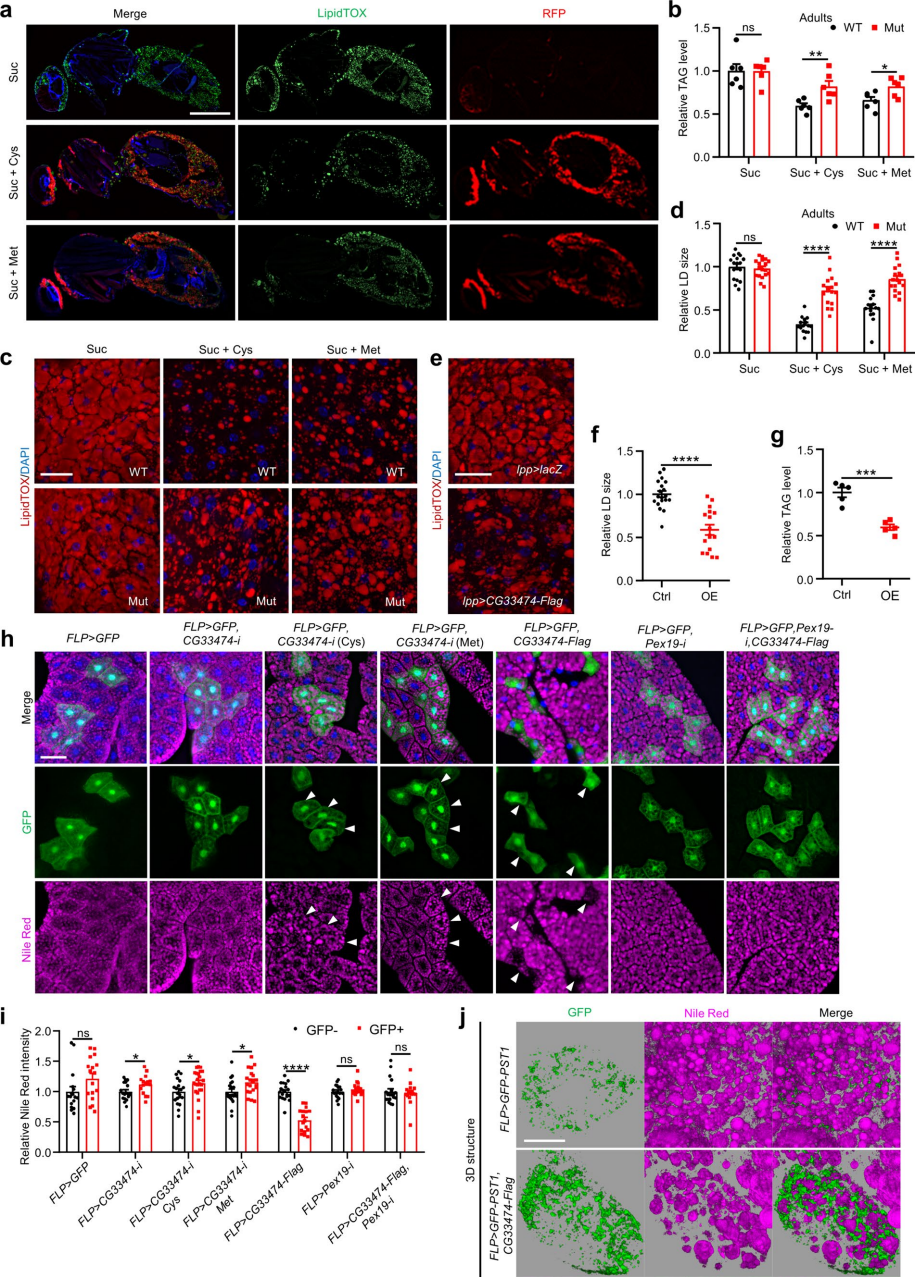
*CG33474* is required for cysteine- and methionine-induced fat loss. **a** Images depicting longitudinal sections of *CG33474-Gal4>UAS-RFP* female flies 36 h following the designated treatments. Suc: 5% sucrose; Suc + Cys: 5% sucrose + 25 mM cysteine; Suc + Met: 5% sucrose + 25 mM methionine. DAPI (blue) labeled nuclei. LipidTOX (green) indicated neutral lipids. Scale bar: 500 μm. **b** TAG levels in female flies 36 h following the designated treatments. Suc: 5% sucrose; Suc + Cys: 5% sucrose + 25 mM cysteine; Suc + Met: 5% sucrose + 25 mM methionine. *w*^*1118*^ and *CG33474* homozygous mutants were used as WT (wild-type) and Mut (mutant), respectively. Data were normalized to protein concentrations. *n* = 6. **c** Lipid staining on female flies 36 h following the designated treatments. Suc: 5% sucrose; Suc + Cys: 5% sucrose + 25 mM cysteine; Suc + Met: 5% sucrose + 25 mM methionine. *w*^*1118*^ and *CG33474* homozygous mutants were used as WT and Mut, respectively. DAPI (blue) labeled nuclei. LipidTOX (red) indicated neutral lipids. Scale bar: 20 μm. **d** Relative size of LDs in **c**. From left to right: 18, 18, 15, 17, 15, and 17 views. **e** Lipid staining on female flies of the designated genotypes. DAPI (blue) labeled nuclei. LipidTOX (red) indicated neutral lipids. Scale bar: 20 μm. **f** Relative size of LDs in **e**. Ctrl: *lpp-Gal4>UAS-lacZ*; OE: *lpp-Gal4>UAS-CG33474-Flag*. From left to right: 19 and 17 views. **g** TAG levels in female flies of the designated genotypes. Ctrl: *lpp-Gal4>UAS-lacZ*; OE: *lpp-Gal4>UAS-CG33474-Flag*. Data were normalized to protein concentrations. *n* = 5. **h** Lipid staining of fat bodies from 3rd instar larvae of the designated genotypes using Nile Red. DAPI (blue) labeled nuclei. Clones were labeled by GFP (green). Nile Red (magenta) indicated neutral lipids. Arrows indicated fat cells expressing clones. Larvae in the 3rd and 4th columns were treated with 25 mM cysteine (Cys) and 25 mM methionine (Met), respectively, for 36 h. Scale bar: 50 μm. **i** Relative fluorescence intensities of Nile Red in **h**. From left to right: 17, 17, 18, 17, 20, 23, 22, 20, 20, 17, 19, 19, 19, and 16 cells. **j** Representative 3D structural images of Nile Red-stained fat bodies from 3rd instar larvae of the indicated genotypes. Nile Red (magenta) indicated neutral lipids. Scale bar: 25 μm. Two-tailed Student’s *t* test was performed. * *p* < 0.05; ** *p* < 0.01; *** *p* < 0.001; **** *p* < 0.0001; ns, not significant

As HPD primarily enhances peroxisome levels in the adipose tissues, we examined whether cysteine- and methionine-induced *CG33474* expression exhibits adipose specificity. Given that cysteine, a semi-essential amino acid derived from the essential amino acid methionine via the transsulfuration pathway [29], exhibits similar effects to methionine in promoting *CG33474* expression, we initially focused on cysteine for preliminary testing. The intensity of red fluorescent protein (RFP) in the adipose tissues of *CG33474-Gal4>UAS-RFP* female flies increased significantly after a 36-h cysteine feeding, indicative of the fact that cysteine-induced *CG33474* expression is predominantly localized to adipose tissues (Fig. S7a–c). This finding was paralleled in *CG33474-Gal4>UAS-RFP* larvae, where cysteine-induced *CG33474* expression was predominantly enriched in the larval fat body (Fig. S7d, e). To solidify this observation, we examined the colocalization of *CG33474* with adipose tissues using *CG33474-Gal4>UAS-RFP* female flies fed with cysteine or methionine for 36 h. Tissue section results revealed that CG33474 proteins co-localized with LipidTOX, a maker of neutral lipids (Fig. 5a; Fig. S7f). Altogether, cysteine- and methionine-induced *CG33474* expression is primarily in the adipose tissues.

### *CG33474* is required for cysteine- and methionine-induced fat loss

In the course of investigating *CG33474* expression patterns using LipidTOX, we noticed a significant decline in lipid contents in flies fed with cysteine or methionine (Fig. 5a). This observation prompted us to explore whether cysteine and methionine regulate lipid metabolism in a *CG33474-*dependent manner. We first confirmed that *w*^*1118*^ female flies fed with a 36-h cysteine or methionine diet exhibited lower lipid contents, as evidenced by measuring their whole-body triglyceride (TAG) levels, an indicator of lipid storage (Fig. S8a, b). Remarkably, *CG33474* mutant female flies were protected from cysteine- and methionine-induced decrease in TAG levels (Fig. 5b; Fig. S8c) and lipid droplets (LDs) size (Fig. 5c, d), suggesting that cysteine- and methionine-triggered fat loss requires *CG33474*. Similarly, in the larval phase, *CG33474* mutants also attenuated cysteine- and methionine-induced TAG reduction (Fig. S8d, e). Moreover, fat body-specific overexpression of *CG33474* (*ppl-Gal4>UAS-CG33474-Flag*) of female flies led to a reduced LD size (Fig. 5e, f) and a diminished TAG level (Fig. 5g; Fig. S8f) compared to driver control flies (*ppl-Gal4>UAS-lacZ*).

We further utilized the FLP-out technique to clonally decrease or increase *CG33474* expression in 3rd instar larval fat bodies and visualized LDs through Nile Red staining. Knockdown of *CG33474* led to slightly elevated levels of neutral lipids within adipocytes compared to control clones (Fig. 5h, i). Additionally, when *CG33474* was knocked down and simultaneously treated with cysteine or methionine for a duration of 36 h also increased neutral lipid levels (Fig. 5h, i), corroborating that cysteine- and methionine-induced lipids decrease requires *CG33474*. Meanwhile, *CG33474*-expressing clones significantly reduced neutral lipid levels within adipocytes compared to control clones, suggesting that *CG33474* stimulates the breakdown of neutral lipids in a cell-autonomous manner (Fig. 5h, i).

Next, we constructed a *Drosophila* line (*FLP>UAS-GFP-PTS1*, *UAS-CG33474-Flag*) that concurrently overexpressed *GFP-PTS1* and *CG33474*. The results demonstrated that overexpressing *CG33474* not only resulted in an elevation in peroxisome levels and a reduction in lipid contents but also facilitated a tight association between LDs and peroxisomes (Fig. 5j; Videos 1, 2), indicating that *CG33474-*induced fat loss might depend on the biogenesis of peroxisomes. To test this hypothesis, we generated a *Drosophila* strain (*FLP>UAS-CG33474-Flag*, *UAS-Pex19-RNAi*) that simultaneously overexpressed *CG33474* and downregulated *Pex19*, a crucial peroxisome biogenesis factor [30]. We successfully gained a *Drosophila* Pmp70 antibody, which performed effectively as determined by the co-localization with the green fluorescence emanating from the *Ubi-GFP-PTS1* line (Fig. S8g). Then, we employed this Pmp70 antibody to confirm that the knockdown of *Pex19* significantly inhibited peroxisome biogenesis (Fig. S8h). Additionally, Nile Red staining revealed that knockdown of *Pex19* did not impact neutral lipid levels (Fig. 5h, i). Subsequently, we visualized LDs within the fat bodies of 3rd instar larval from the *FLP>UAS-CG33474-Flag*, *UAS-Pex19-RNAi* strain using Nile Red staining. The results demonstrated that suppressing peroxisome biogenesis inhibited *CG33474*-induced fat loss (Fig. 5h, i), indicating that *CG33474*-induced fat loss is dependent on peroxisome biogenesis.

Next, we explored the possible effects of other signaling pathways on *CG33474*-induced fat loss. The Nile Red staining results revealed that the knockdown of *bmm* (a triglyceride lipase), or ROS scavengers such as *Sod1* (located in the cytoplasm) and *Sod2* (located in the matrix), failed to abrogate the effect of *CG33474* in reducing neutral lipid levels (Fig. S8i, j). This finding suggested that *CG33474*-induced fat loss is independent of *bmm*-mediated lipid metabolism process or the *Sod*-regulated ROS pathway.

### Roles of TOR signaling in cysteine- and methionine-induced *CG33474* expression

**Fig. 6:**
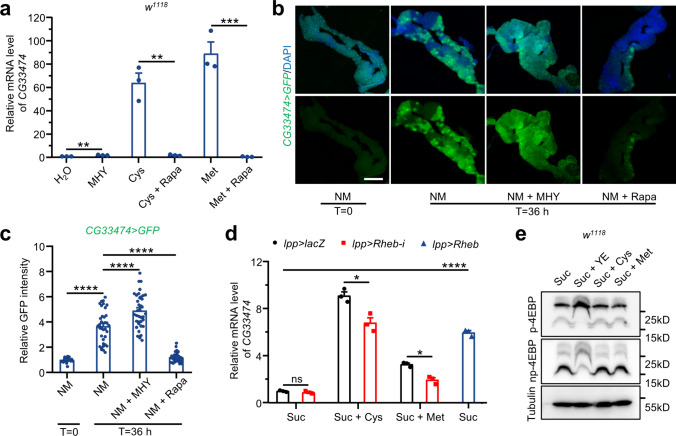
Roles of TOR signaling in cysteine- and methionine-induced *CG33474* expression. **a** Relative *CG33474* mRNA levels in *w*^*1118*^ female flies 36 h following the designated treatments. Ctrl: H_2_O; MHY: 100 μΜ MHY1485; Cys: 25 mM cysteine; Cys + Rapa: 25 mM cysteine + 200 μΜ rapamycin; Met: 25 mM methionine; Met + Rapa: 25 mM methionine + 200 μΜ rapamycin. *n* = 3. **b** Fluorescence microscopy images of *CG33474-Gal4>UAS-GFP* 3rd instar larval fat bodies cultured under the indicated conditions. NM: normal Schneider medium; NM + MHY: normal Schneider medium containing 10 μΜ MHY1485; NM + Rapa: normal Schneider medium containing 10 μΜ rapamycin. DAPI (blue) labeled nuclei. Scale bar: 100 μm. T: time. **c** Relative fluorescence intensities of GFP in **b**. From left to right: 21, 36, 37, and 33 views. **d** Relative *CG33474* mRNA levels in female flies of the designated genotypes after 36 h of feeding with the indicated treatments. Suc: 5% sucrose; Suc + Cys: 5% sucrose + 25 mM cysteine; Suc + Met: 5% sucrose + 25 mM methionine. *n* = 3. **e** Western blotting showing the levels of p-4EBP and np-4EBP in *w*.^*1118*^ female flies under the indicated treatments for 36 h. Suc: 5% sucrose; Suc + YE: 5% sucrose + 30% yeast extract; Suc + Cys: 5% sucrose + 25 mM cysteine; Suc + Met: 5% sucrose + 25 mM methionine. Two-tailed Student’s *t* test (**a**, **d**) or one-way ANOVA (**c**) were performed. * *p* < 0.05; ** *p* < 0.01; *** *p* < 0.001; **** *p* < 0.0001

Since a recent study identified that cystine, the oxidized derivative of cysteine, activates TOR signaling in the fat body of fasted *Drosophila* [31], we conjectured that TOR signaling might be indispensable for cysteine- and methionine-induced *CG33474* expression. To probe this hypothesis, we fed *w*^*1118*^ female flies with different compounds for 36 h and assessed the mRNA levels of *CG33474* by RT-qPCR. Treatment with the classic TOR allosteric inhibitor rapamycin [32] effectively attenuated the elevation of *CG33474* induced by cysteine and methionine (Fig. 6a). Furthermore, exposure to the cell-permeable TOR activator, MHY1485 [33], resulted in a slight increase in *CG33474* expression (Fig. 6a).

To further elucidate the roles of TOR signaling in *CG33474* expression, we ex vivo cultured fat bodies isolated from *CG33474-Gal4>UAS-GFP* 3rd instar larvae for a duration of 36 h with different chemicals. The results showed that incubating the fat bodies in Schneider medium significantly induced *CG33474* expression as determined by the heightened green fluorescence intensity, and the addition of MHY1485 further potentiated this upregulation (Fig. 6b, c). On the other hand, rapamycin substantially curtailed the upregulation of *CG33474* compared to Schneider medium vehicle exposure (Fig. 6b, c).

Ultimately, we employed a genetic approach to substantiate the preceding findings. We collected flies that had been subjected to various diets and analyzed the mRNA levels of *CG33474* using RT-qPCR. The results demonstrated that the inhibition of the TOR pathway by knocking down *Rheb* in the fat bodies (*lpp-Gal4>UAS-Rheb-RNAi*) effectively diminished cysteine- and methionine-induced *CG33474* expression (Fig. 6d). Conversely, overexpression of *Rheb* in the fat bodies (*lpp-Gal4>UAS-Rheb*) to activate the TOR pathway was adequate to trigger *CG33474* expression even in the absence of cysteine and methionine (Fig. 6d). Together, these data suggested that TOR signaling is required for cysteine- and methionine-induced *CG33474* expression.

In addition, we observed that while TOR signaling activity was significantly increased in flies fed with HPD, as determined by elevated eIF4E-binding protein (4EBP) phosphorylation level, it remained unchanged in cysteine- and methionine-treated flies (Fig. 6e). These data showed that while TOR activity promotes *CG33474* expression, and a basal level of TOR activity is required for the induction of *CG33474*. However, an increase in TOR signaling is not required for the upregulation of *CG33474*. Taken together, our data suggested that TOR serves as a regulator, yet some unidentified amino acid sensing pathway might be directly responsible for the induction of *CG33474*.

### The functions of *PEX11G* are evolutionarily conserved

Finally, we investigated whether the induction of *CG33474*/*PEX11G* is evolutionarily conserved. Mammalian HEK293T cells were cultured in the normal medium supplemented with varying concentrations of cysteine or methionine (with 25 mM cysteine excluded due to its severe cytotoxic effect). Following a 36-h incubation, we quantified *PEX11G* mRNA levels using RT-qPCR. The results revealed that supplementation with 15 mM cysteine or methionine moderately promoted *PEX11G* expression in HEK293T cells (Fig. S9a). Analogous to the findings in *Drosophila*, MHY1485 also increased *PEX11G* expression, while rapamycin attenuated cysteine- and methionine-induced *PEX11G* expression in HEK293T cells (Fig. S9b). However, it is noteworthy that the induction of *PEX11G* by cysteine and methionine in HEK293T cells was less prominent than that in flies. It is possible that stronger effects might be triggered in certain adipocyte cell lines as cysteine- and methionine-induced *CG33474* expression is primarily in the adipose tissues in flies.

**Fig. 7:**
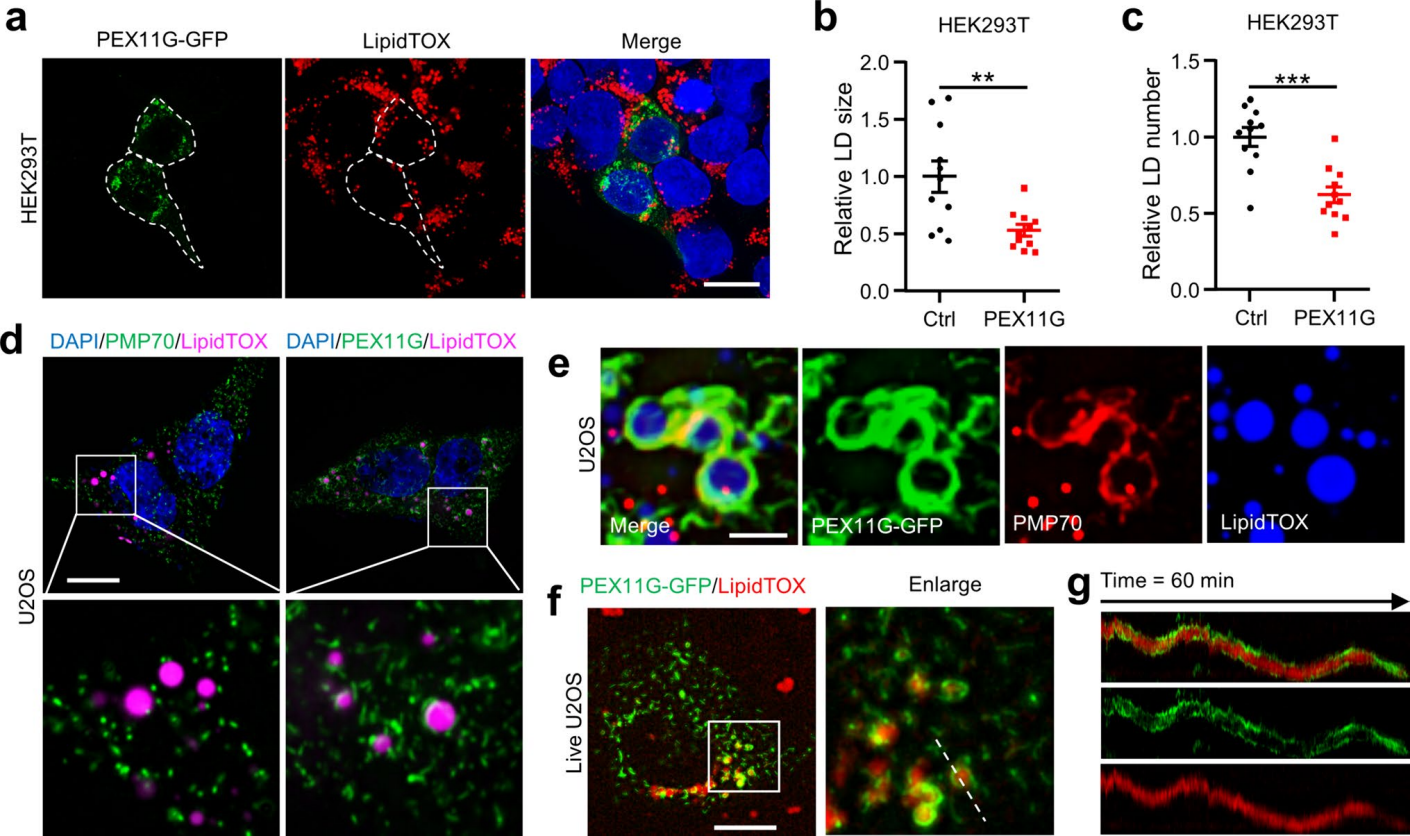
The functions of *PEX11G* are evolutionarily conserved. **a** Fluorescence microscopy images of HEK293T cells transfected with PEX11G-GFP (green) encoding plasmid and treated with 100 μΜ OA. DAPI (blue) labeled nuclei. LipidTOX (red) indicated neutral lipids. Dotted lines marked the outlines of *PEX11G*-expressing cells. Scale bar: 20 μm. Relative size (**b**) and number (**c**) of LDs between control (Ctrl) and *PEX11G*-expressing cells (PEX11G) in **a**. *n* = 11. **d** Fluorescence microscopy images of peroxisome-LD contacts in U2OS cells transfected with PEX11G-GFP (green) encoding plasmid or not and treated with 100 μΜ OA. DAPI (blue) labeled nuclei. LipidTOX (magenta) indicated neutral lipids. Scale bar: 10 μm. **e** Fluorescence microscopy images of peroxisome-LD contacts in U2OS cells transfected with PEX11G-GFP (green) encoding plasmid and treated with 100 μΜ OA. The boxed areas were enlarged to the lower panel. LipidTOX (blue) indicated neutral lipids. PMP70 (red) indicated peroxisomes. Scale bar: 2 μm. **f** A live cell image depicting U2OS cells transfected with PEX11G-GFP (green) encoding plasmid. The boxed area was enlarged to the right. LipidTOX (red) indicated neutral lipids. Scale bar: 10 μm. **g** Fluorescence profiles derived from the indicated dotted line scan in **f** for 60 min. Two-tailed Student’s *t* test was performed. ** *p* < 0.01; *** *p* < 0.001

To assess whether the function of *PEX11G* in lipid metabolism is evolutionarily conserved, we transfected HEK293T cells with a plasmid encoding PEX11G-GFP and subsequently evaluated the LD index. *PEX11G*-expressing cells exhibited a decrease in both the size and number of LDs (Fig. 7a–c), indicating an accelerated rate of lipid breakdown.

Next, we investigated the interplay between *PEX11G* and LDs, as previous studies reported that peroxisomes and LDs are in physical contact within cells [34]. We used the U2OS cell line, which is frequently employed for live imaging of organelle dynamics due to its flat structure. We observed that, in un-transfected U2OS cells stained with PMP70 antibody, peroxisomes exhibited little association with LDs (Fig. 7d). In contrast, peroxisomes in *PEX11G*-expressing U2OS cells showed a strong association with LDs (Fig. 7d). Furthermore, this interaction is not caused by mis-localization of PEX11G-GFP onto the surface of LDs, since the identical PEX11G-GFP proteins colocalized with the peroxisome marker PMP70 (Fig. 7e), suggesting that *PEX11G* facilitates the association between peroxisomes and LDs. A similar association between peroxisomes and LDs triggered by *PEX11G* was also observed in HEK293T cells, albeit to a lesser extent (Fig. S9c; Videos 3, 4). This finding is consistent with our previous discovery that peroxisomes exhibit increased attachment to LDs in larval fat cells (Fig. 5j). The peroxisomes-LDs interaction is further supported by the live-imaging experiment, as PEX11G-tagged peroxisomes tightly associated with LDs for more than 1 h (Fig. 7f, g; Video 5). Altogether, these data strongly suggested that *PEX11G* promotes inter-organelle contacts between peroxisomes and LDs, potentially accounting for *PEX11G*-induced fat loss.

Then, we investigate whether *PEX11G* regulates peroxisomal biosynthesis, import, and assembly. To this end, we overexpressed PEX11G-GFP fusion proteins in HEK293T cells and conducted western blotting to assess the levels of key peroxisomal proteins. The results revealed that *PEX11G* did not affect proteins that govern peroxisomal morphology, including Dynamin-related protein 1 (Drp1) and Fission factor 1 (Fis1) [30] (Fig. S10a). Additionally, the protein levels of PMP70, PEX5, and PEX19 remained unaltered in *PEX11G-*expressing cells (Fig. S10b). These results proved that *PEX11G* does not modulate the biosynthesis, import, or assembly of peroxisomes. Furthermore, the protein levels of Sod1, Catalase, and ACOX1 (a rate-limiting enzyme for peroxisomal β-oxidation [35]) remained unchanged in *PEX11G-*expressing cells (Fig. S10b). Additionally, *PEX11G* expression did not colocalize with PEX5, PEX19, or Catalase in HEK293T cells (Fig. S10c–e).

Given the crucial role of peroxisomes in the detoxification of ROS [36], we investigated the potential impact of *PEX11G* on ROS levels. Dihydroethidium (DHE) staining revealed that overexpression of *PEX11G* reduced ROS levels in HEK293T cells (Fig. S11a, b). Similarly, overexpressing *CG33474* in *Drosophila* also resulted in a decrease in ROS levels, as demonstrated by DHE staining (Fig. S11c, d). Together, our data showed that overexpression of *CG33474* or *PEX11G* can mitigate ROS production in *Drosophila* and mammalian cells, respectively. Moreover, fat body-specific overexpression of *CG33474* (*ppl-Gal4>UAS-CG33474-Flag*) of larvae did not alter lipid peroxidation levels in the fat bodies compared to driver control larval fat bodies (*ppl-Gal4>UAS-lacZ*), as determined by C11 BODIPY 581/591 probe (Fig. S11e, f).

## Discussion

Over the previous three decades, the incidence of obesity has escalated dramatically, almost tripling in prevalence and continuing to rise. Alarmingly, nearly one-third of the world’s population is currently overweight. Obesity poses a myriad of health issues and imposes a substantial economic burden on individuals and society [37]. Although clinical reports [38] and different model organisms, including mice and fruit flies [39, 40], have demonstrated that high-protein diet (HPD) is a practical weight control approach, the underlying molecular mechanisms behind HPD-induced weight loss remain elusive. A recently published study uncovered that dietary cysteine in HPD effectively reduces fat storage in both fruit flies and mice. Cysteine promotes the production of neuropeptide FMRFa, which serves dual functions: it stimulates the activity of PKA and lipase to promote the breakdown of fat, and it restrains food intake by suppressing appetite perception [40]. In this paper, we initially discovered that HPD significantly elevates peroxisome levels specifically in the adipose tissues of *Drosophila*, a phenomenon credited to the presence of cysteine and methionine. As the study processed, we demonstrated that dietary cysteine and methionine lowered lipid storage in fruit flies and proposed a different mechanism: cysteine and methionine trigger the expression of a peroxisomal gene *CG33474*, which facilitates the breakdown of fat in a peroxisome-independent mechanism.

The *PEX11* family of membrane proteins is ubiquitously present across fungi, plant, and mammalian species, exerting a pivotal role in regulating peroxisome morphology, size, and number [41, 42]. The mammalian genome encodes three *PEX11* members: *PEX11A*, *PEX11B*, and *PEX11G* [43]. In *Drosophila*, there are three genes in the *Pex11* family, including *Pex11ab*, *Pex11c*, and *CG33474*. The fly *Pex11ab* is an ortholog to human *PEX11A*/*B*, while both *Pex11c* and *CG33474* exhibit homology to human *PEX11G* [44]. A previous study discovered that heterologous PEX11 proteins localize to peroxisomes in both human cells and plants, and ectopic expression of PEX11 proteins leads to peroxisome proliferation in these organisms [28]. Our study confirmed that both the *Drosophila* CG33474 protein and its human counterpart PEX11G protein localize to peroxisomes, and overexpression of *CG33474*/*PEX11G* increases the size of peroxisomes. Moreover, we discovered that TOR signaling is required for cysteine- and methionine-induced fly *CG33474* and human *PEX11G* expression. Therefore, our findings provide a new molecular link between specific amino acids and peroxisome dynamics.

This study has several limitations that we would like to discuss. Firstly, given that both cysteine and methionine elicited similar effects on elevating peroxisome levels and stimulating *CG33474* expression, we hypothesize that they might operate through a shared metabolite. We intend to delve into this hypothesis in future investigations. Secondly, we observed an augmentation in the interaction between peroxisomes and LDs in *PEX11G*-expressing cells. We speculate that the possible reason why PEX11G promotes lipolysis is: PEX11G promotes peroxisomes and LDs interaction. Nevertheless, the specific mechanism underlining this process is yet to be investigated. Thirdly, apart from its impacts on peroxisome dynamics and lipid metabolism, we also observed that *PEX11G* can mitigate ROS production in both *Drosophila* and mammals. However, as this aspect was not the primary focus of the present work, we did not explore it exhaustively. We intend to investigate the role of *PEX11G* in ROS regulation in future studies. Lastly, the response of *PEX11G* to cysteine and methionine is less evident in HEK293T cells, possibly because the induction of *CG33474* by these amino acids primarily concentrated in the adipose tissues of *Drosophila*. Hence, we speculated that the induction of *PEX11G* by cysteine and methionine might be more pronounced in adipocyte cell lines. Given that our laboratory predominantly utilizes *Drosophila* as a model organism and lacks the necessary experimental conditions for cell studies, we plan to collaborate with other laboratories in the future to address this issue.

In conclusion, we revealed that HPD (cysteine and methionine being key factors) induces the expression of the peroxisomal gene *CG33474* in the adipose tissue, thereby elevating the expression of peroxisomes. Furthermore, we demonstrated that *CG33474* overexpression leads to increased peroxisome size and reduced neutral lipid contents. In summary, our findings present a novel mechanism underlying HPD-induced fat loss and offer a promising therapeutic target for treating obesity.

### Supplementary Information

Below is the link to the electronic supplementary material.

Supplementary file1 (DOCX 39921 KB)

Supplementary file2 (MP4 796 KB)

Supplementary file3 (MP4 799 KB)

Supplementary file4 (MP4 1540 KB)

Supplementary file5 (MP4 1551 KB)

Supplementary file6 (MP4 3933 KB)

## Data Availability

The data presented in this study are available to the corresponding author upon request. The RNA-Seq data generated in this work have been deposited in the National Center for Biotechnology Information (NCBI) under the accession number PRJNA1013606.
