# Supplementary material for: Dietary cysteine and methionine promote peroxisome elevation and fat loss by induction of CG33474 expression in Drosophila adipose tissue
Source: Cell Mol Life Sci. 2024 Apr 22;81(1):190. doi: 10.1007/s00018-024-05226-y (PMC11035426; doi:10.1007/s00018-024-05226-y)
Supplement: Supplementary file 1 — Supplementary file1 (DOCX 39921 KB) [file 18_2024_5226_MOESM1_ESM.docx]

**
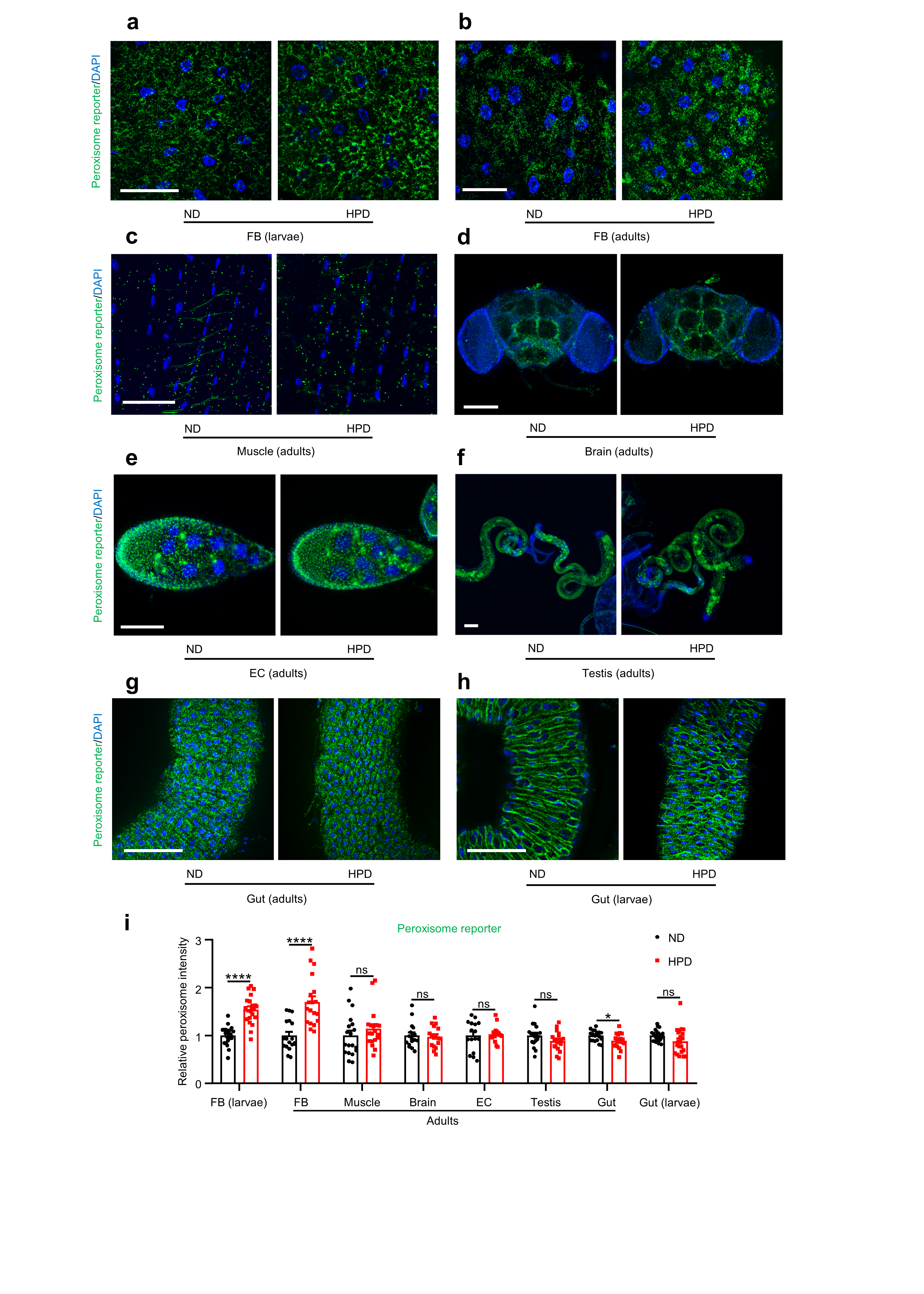
Fig.S1** **HPD-induced peroxisome elevation is primarily in the adipose tissues.**

**a-h** The larval fat bodies (**a**), adult fat bodies (**b**), adult muscles (**c**), adult brains (**d**), adult egg chambers (**e**), adult testes (**f**), adult guts (**g**), and larval guts (**h**) were dissected from the *Ubi-GFP-PTS1* line, which had been fed with a 36-hour ND or HPD. ND: normal diet; HPD: high-protein diet. DAPI (blue) labeled nuclei. Scale bars, 100 μm. **i** Relative fluorescence intensities of peroxisomes in (**a-h**). From left to right: 21, 21, 18, 19, 19, 19, 16, 17, 16, 16, 18, 19, 19, 21, 22, and 22 views. Two-tailed Student's *t* test was performed. * *p* < 0.05; **** *p* < 0.0001; ns, not significant.


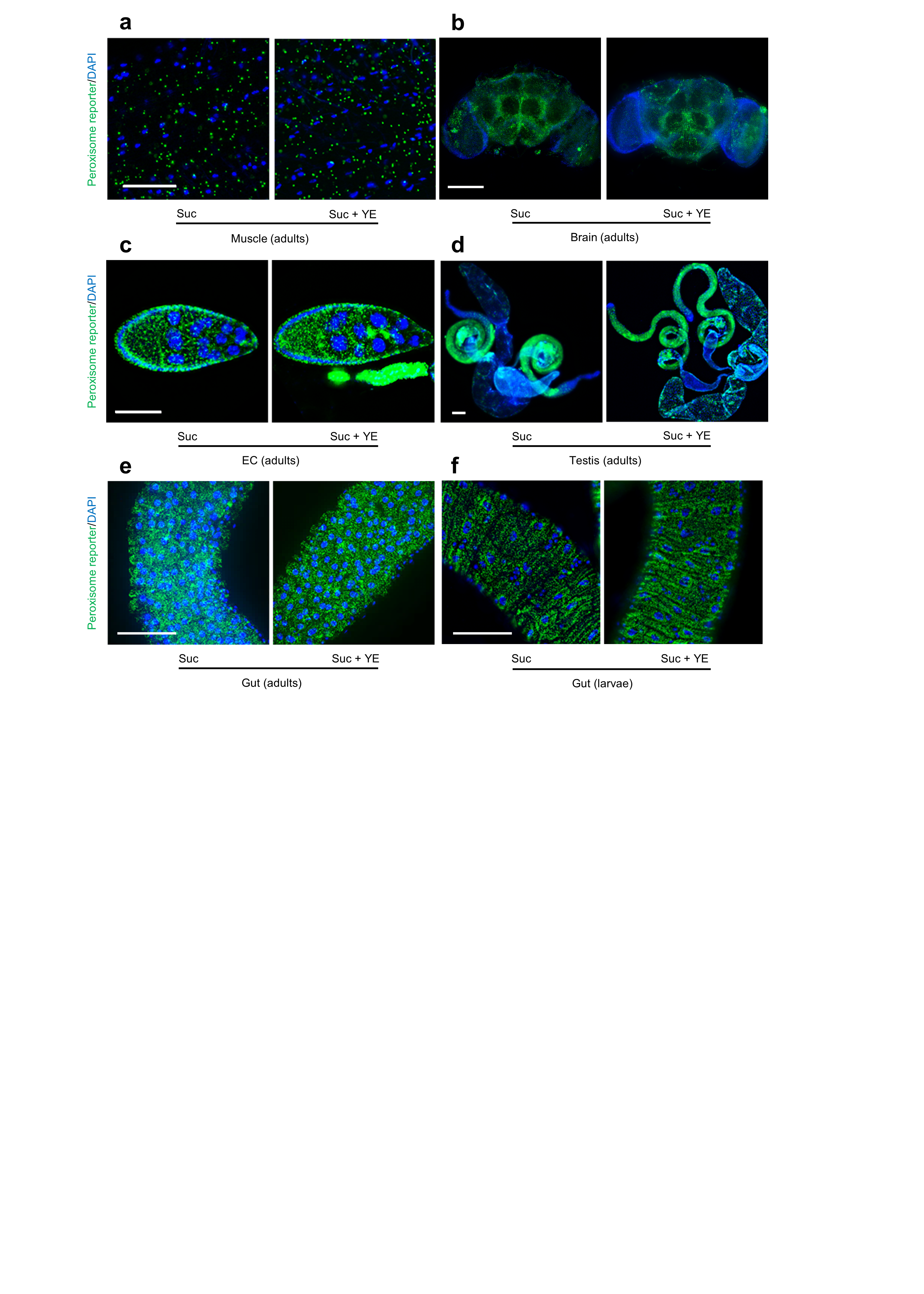
**Fig.S2** **YE-induced peroxisome elevation is primarily in the adipose tissues.**

The adult muscles (**a**), adult brains (**b**), adult egg chambers (**c**), adult testes (**d**), adult guts (**e**), and larval guts (**f**) were dissected from the *Ubi-GFP-PTS1* line, which had been fed with a 36-hour Suc (5% sucrose) or Suc + YE (5% sucrose + 30% yeast extract). DAPI (blue) labeled nuclei. Scale bars, 100 μm.


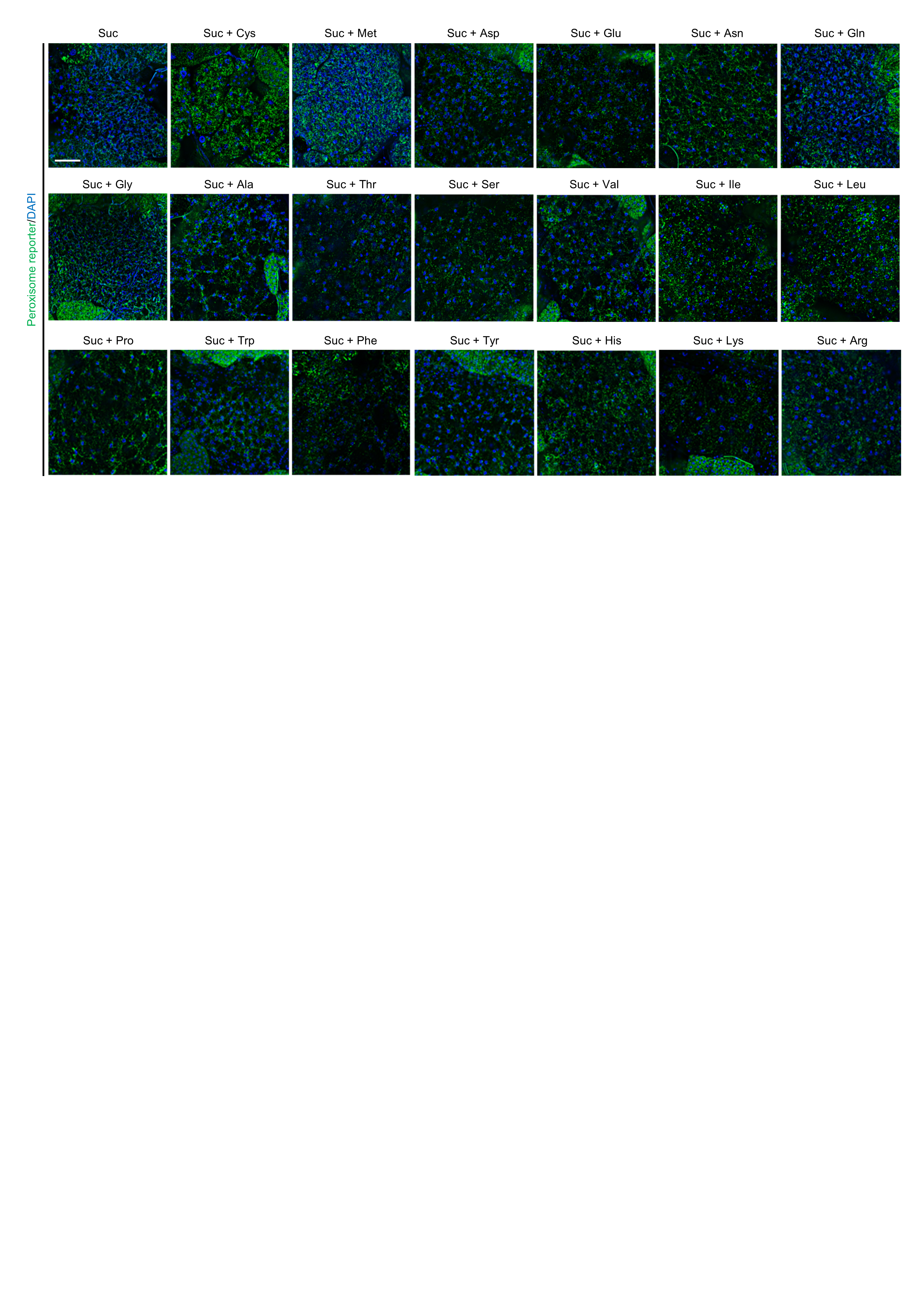
**Fig.S3 Cysteine and methionine are key factors in HPD-induced peroxisome elevation.**

Peroxisome levels in the adipose tissues of *Ubi-GFP-PTS1* female flies 36 h following the indicated treatments. Suc: 5% sucrose; Others: 5% sucrose + 25 mM individual amino acids. Scale bar, 100 μm.


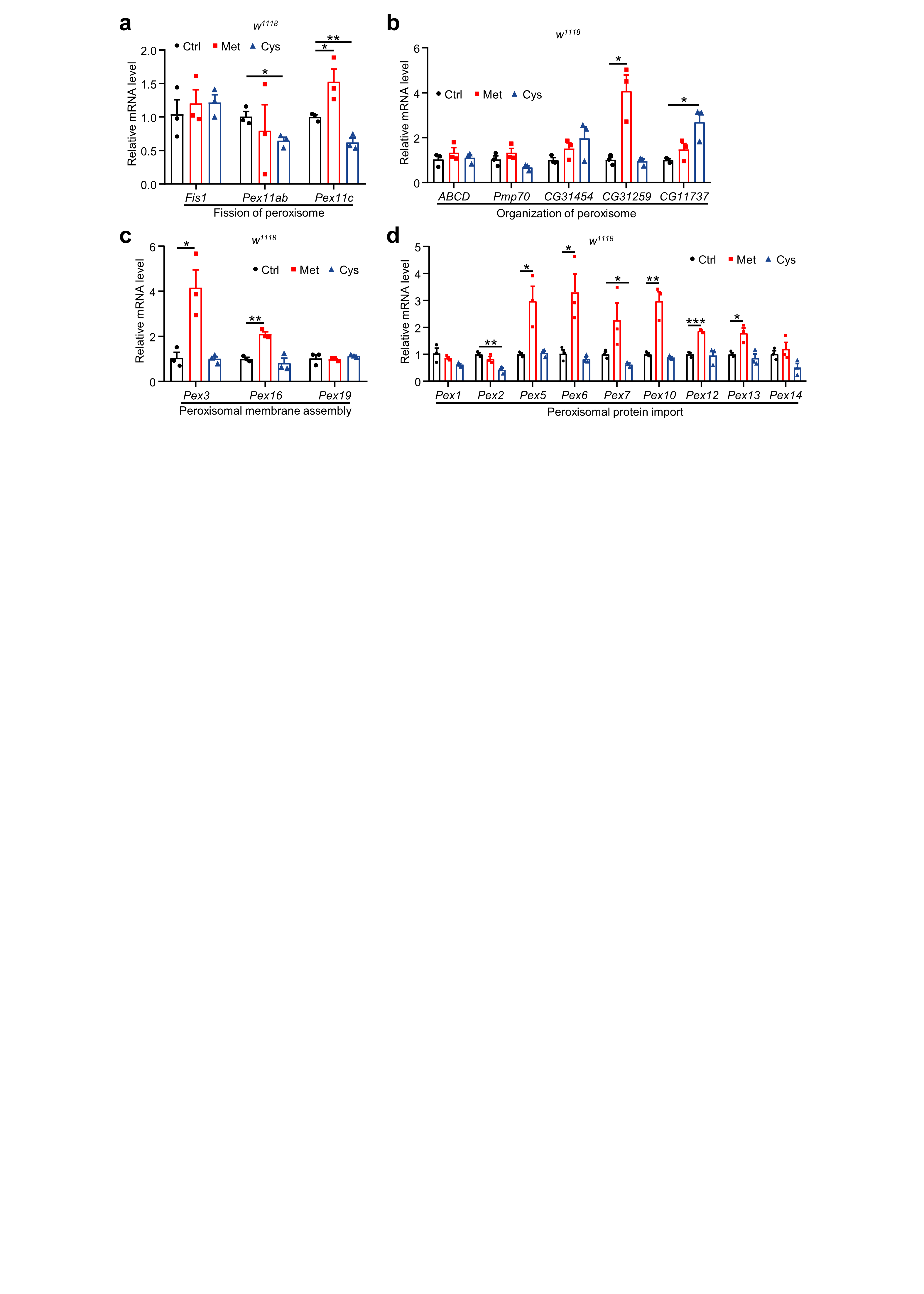
**Fig.S4** **HPD elevates peroxisome levels by triggering *CG33474* expression.**

Relative mRNA levels of genes involved in fission of peroxisome (**a**), organization of peroxisome (**b**), peroxisomal membrane assembly (**c**), and peroxisomal protein import (**d**) in *w^1118^* female flies after being subjected to the indicated treatments for 36 h. Ctrl: 1% agarose; Met: 1% agarose + 25 mM methionine; Cys: 1% agarose + 25 mM cysteine. *n* = 3. Two-tailed Student's *t* test was performed. * *p* < 0.05; ** *p* < 0.01; *** *p* < 0.001.


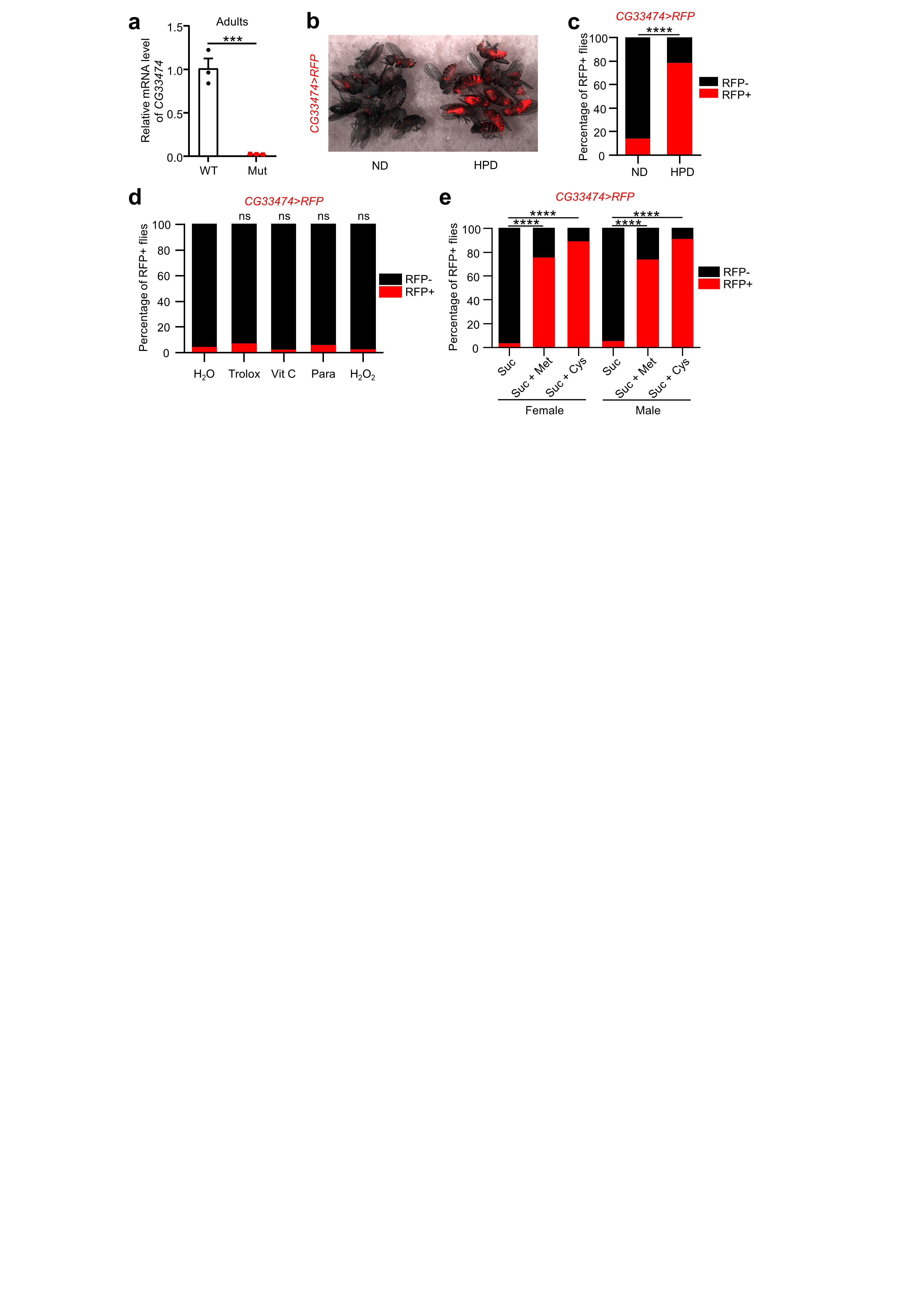
**Fig.S5 The induction of *CG33474* under different conditions.**

**a** Relative *CG33474* mRNA levels in female flies of the designated genotypes. *w^1118^* and *CG33474* homozygous mutants were used as WT and Mut, respectively. *n* = 3. **b**-**c** Represent image (**b**) and quantification (**c,** from left to right: 36 and 37 flies.) of *CG33474-Gal4>UAS-RFP* female flies expressing RFP 36 h following the indicated treatments. ND: normal diet; HPD: high-protein diet. **d** Percentage of *CG33474-Gal4>UAS-RFP* female flies expressing RFP 36 h following the indicated treatments. Trolox: 20 mM Trolox; Vit C: 20 mM Vitamin C; Para: 20 mM paraquat; H_2_O_2_: 5% H_2_O_2_. From left to right: 39, 38, 33, 31, and 32 flies. **e** Percentage of *CG33474-Gal4>UAS-RFP* female and male flies expressing RFP 36 h following the indicated treatments. Suc: 5% sucrose; Suc + Met: 5% sucrose + 25 mM methionine; Suc + Cys: 5% sucrose + 25 mM cysteine. From left to right: 48, 42, 48, 50, 47, and 49 flies. Fisher's Exact Test was performed. **** *p* < 0.0001; ns, not significant.


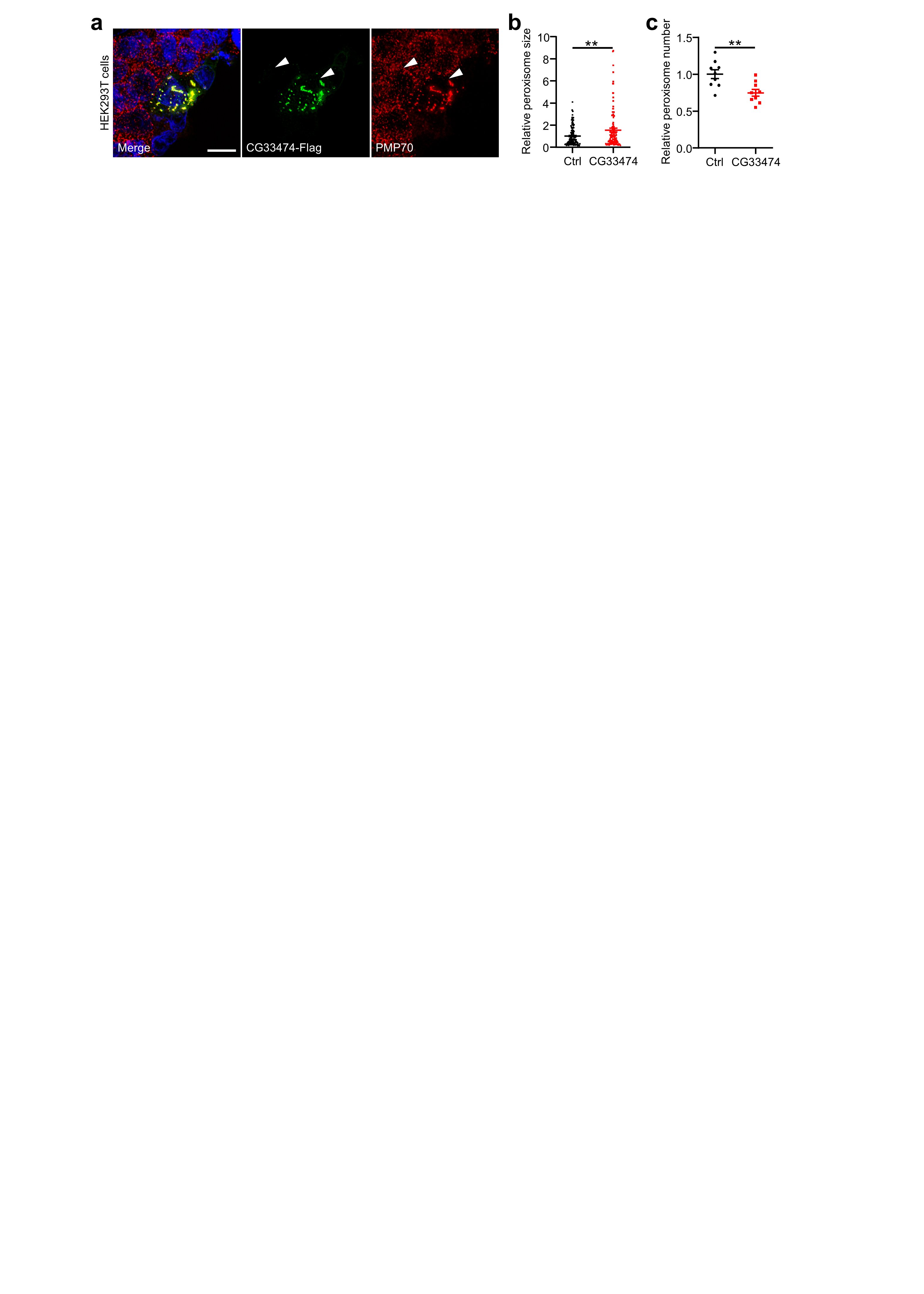
**Fig.S6 CG33474 proteins lead to increased peroxisome size.**

**a** Fluorescence microscopy images of HEK293T cells transfected with CG33474-Flag (green) encoding plasmid. DAPI (blue) labeled nuclei. PMP70 (red) indicated peroxisomes. Arrows indicated a significant difference between control and *CG33474-*expressing cells. Scale bar, 20 μm. **b-c** Relative peroxisome size (**b,** *n* = 10 cells.) and number (**c,** *n* = 10 cells.) between control (Ctrl) and *CG33474* expressing cells (CG33474) in (**a**). Two-tailed Student's *t* test was performed. ** *p* < 0.01.


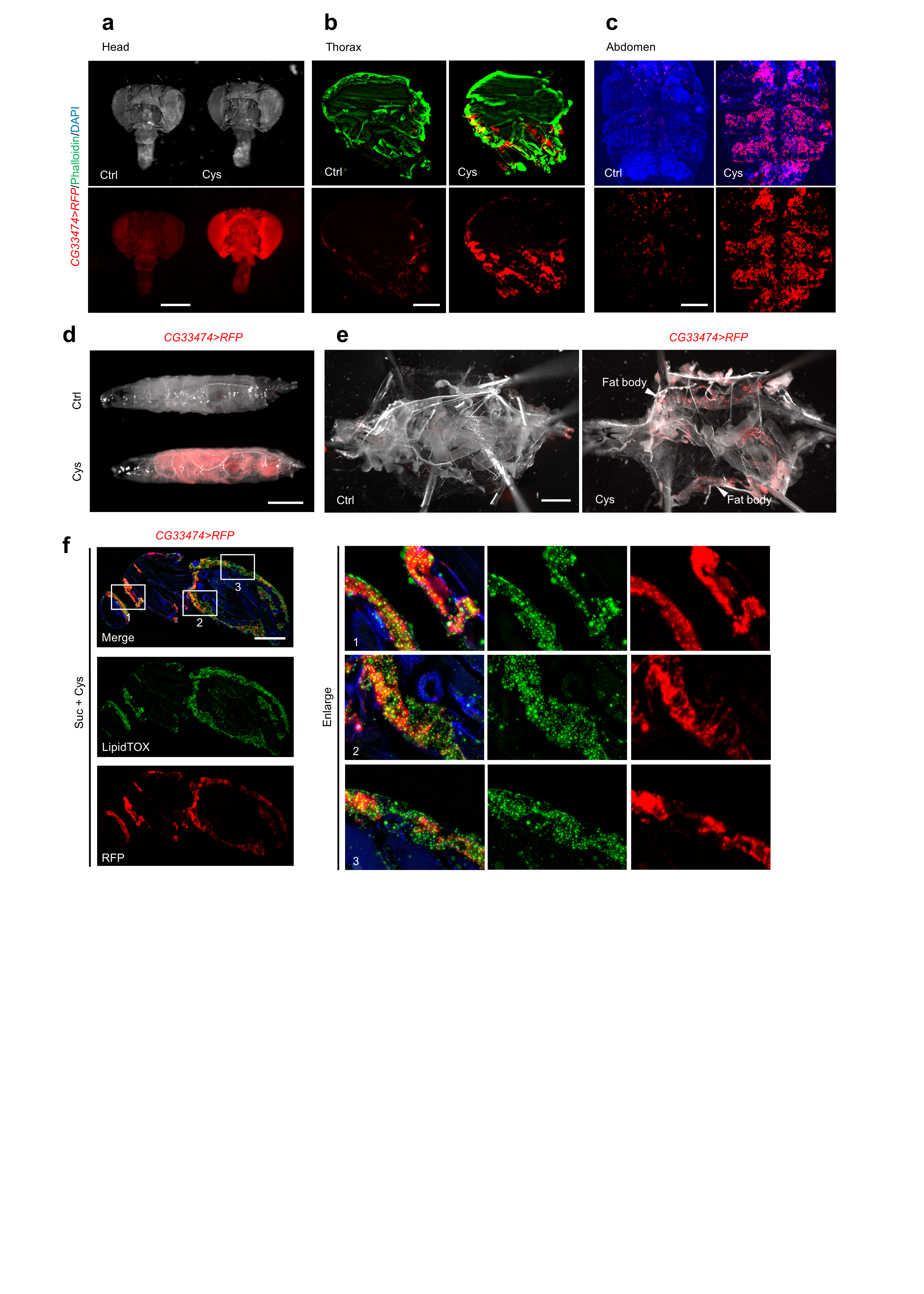
**Fig.S7 Cysteine- and methionine-induced *CG33474* expression is primarily in the adipose tissues.**

**a-c** The heads (**a**), thoraxes (**b**), and abdomens (**c**) were dissected from the *CG33474-Gal4>UAS-RFP* female flies, which had been fed with a 36-hour Ctrl (1% agarose) or Cys (1% agarose + 25 mM cysteine) diets. DAPI (blue) labeled nuclei. Phalloidin (green) indicated filamentous actin. Scale bars, 200 μm. **d** Image of *CG33474-Gal>UAS-RFP* larvae treated with Ctrl (1% agarose) or Cys (1% agarose + 25 mM cysteine) for 36 h. Scale bar, 1 mm. **e** Dissections of *CG33474-Gal4>UAS-RFP* larvae treated with Ctrl (1% agarose) or Cys (1% agarose + 25 mM cysteine) for 36 h. Arrows indicated fat bodies. Scale bar, 1 mm. **f** Images depicting longitudinal sections of *CG33474-Gal4>UAS-RFP* female flies treated with Suc + Cys (5% sucrose + 25 mM cysteine) for 36 h. The boxed areas in (1)-(3) were enlarged to the right. DAPI (blue) labeled nuclei. LipidTOX (green) indicated neutral lipids. Scale bar, 500 μm.

**
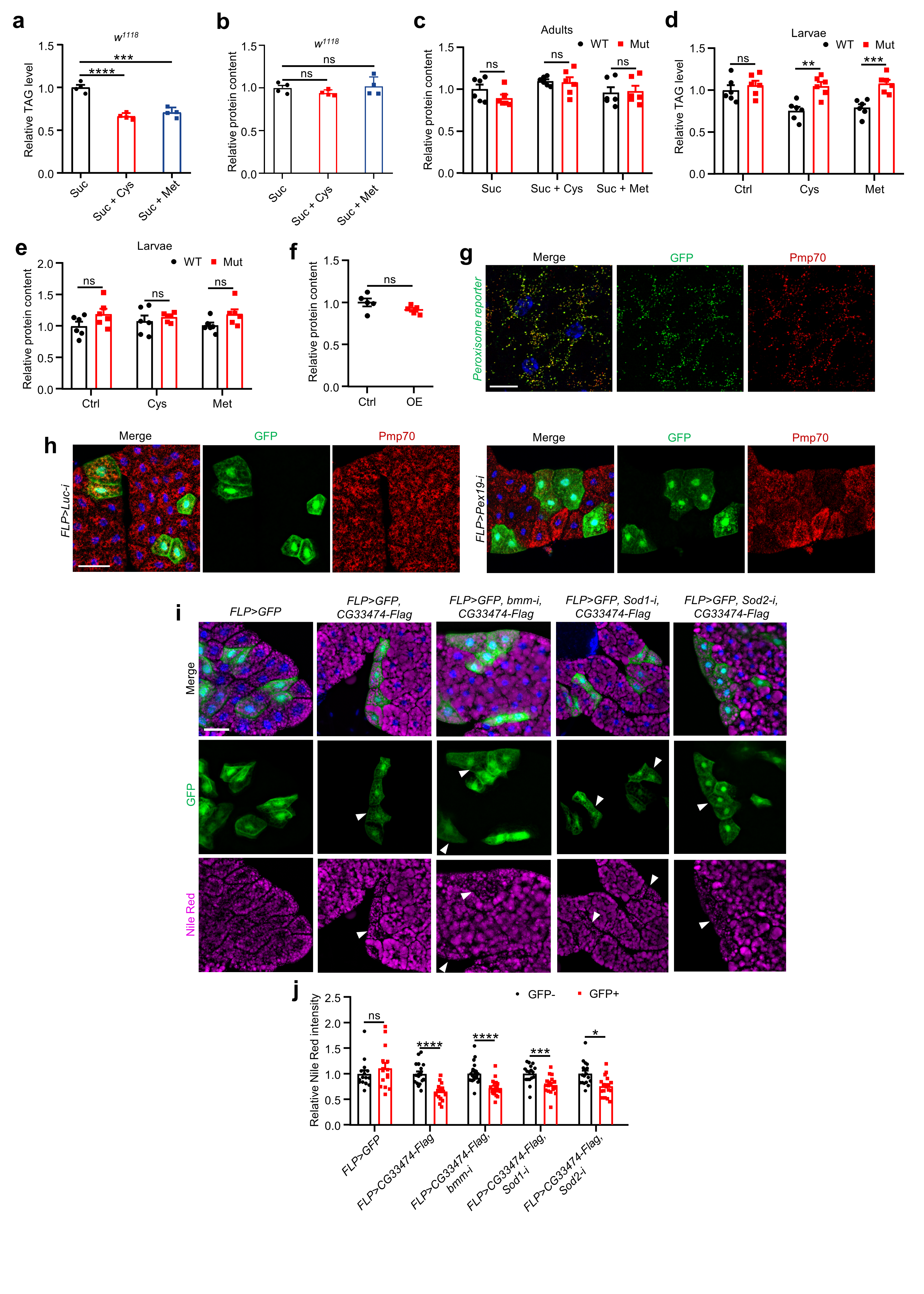
**

**Fig.S8 *CG33474* is required for cysteine- and methionine-induced fat loss.**

**a-b** TAG levels (**a**) and protein contents (**b**) in *w^1118^* female flies 36 h following the designated treatments. Suc: 5% sucrose; Suc + Cys: 5% sucrose + 25 mM cysteine; Suc + Met: 5% sucrose + 25 mM methionine. Data were normalized to protein concentrations. *n* = 4. **c** Protein contents in female flies 36 h following the designated treatments in **Fig. 5b**. **d-e** TAG levels (**d**) and protein contents (**e**) in larvae 36 h following the designated treatments. Ctrl: 1% agarose; Cys: 1% agarose + 25 mM cysteine; Met: 1% agarose + 25 mM methionine. *w^1118^* and *CG33474* homozygous mutants were used as WT and Mut, respectively. Data were normalized to protein concentrations. *n* = 6. **f** Protein contents in female flies of the designated genotypes in **Fig. 5g**. **g** Fluorescence microscopy images of *Ubi-GFP-PTS1* female flies. DAPI (blue) labeled nuclei. Pmp70 (red) indicated peroxisomes. Scale bar, 10 μm. **h** Fluorescence microscopy images of fat bodies from 3^rd^ instar larvae of the designated genotypes. DAPI (blue) labeled nuclei. Clones were labeled by GFP (green). Pmp70 (red) indicated peroxisomes. Scale bar, 100 μm. **i** Lipid staining of fat bodies from 3^rd^ instar larvae of the designated genotypes using Nile Red. DAPI (blue) labeled nuclei. Clones were labeled by GFP (green). Nile Red (magenta) indicated neutral lipids. Arrows indicated fat cells expressing clones. Scale bar, 50 μm. **j** Relative fluorescence intensities of Nile Red in (**i**). From left to right: 15, 15, 19, 17, 22, 18, 17, 18, 18, and 18 cells. Two-tailed Student's *t* test was performed. * *p* < 0.05; ** *p* < 0.01; *** *p* < 0.001; **** *p* < 0.0001; ns, not significant.


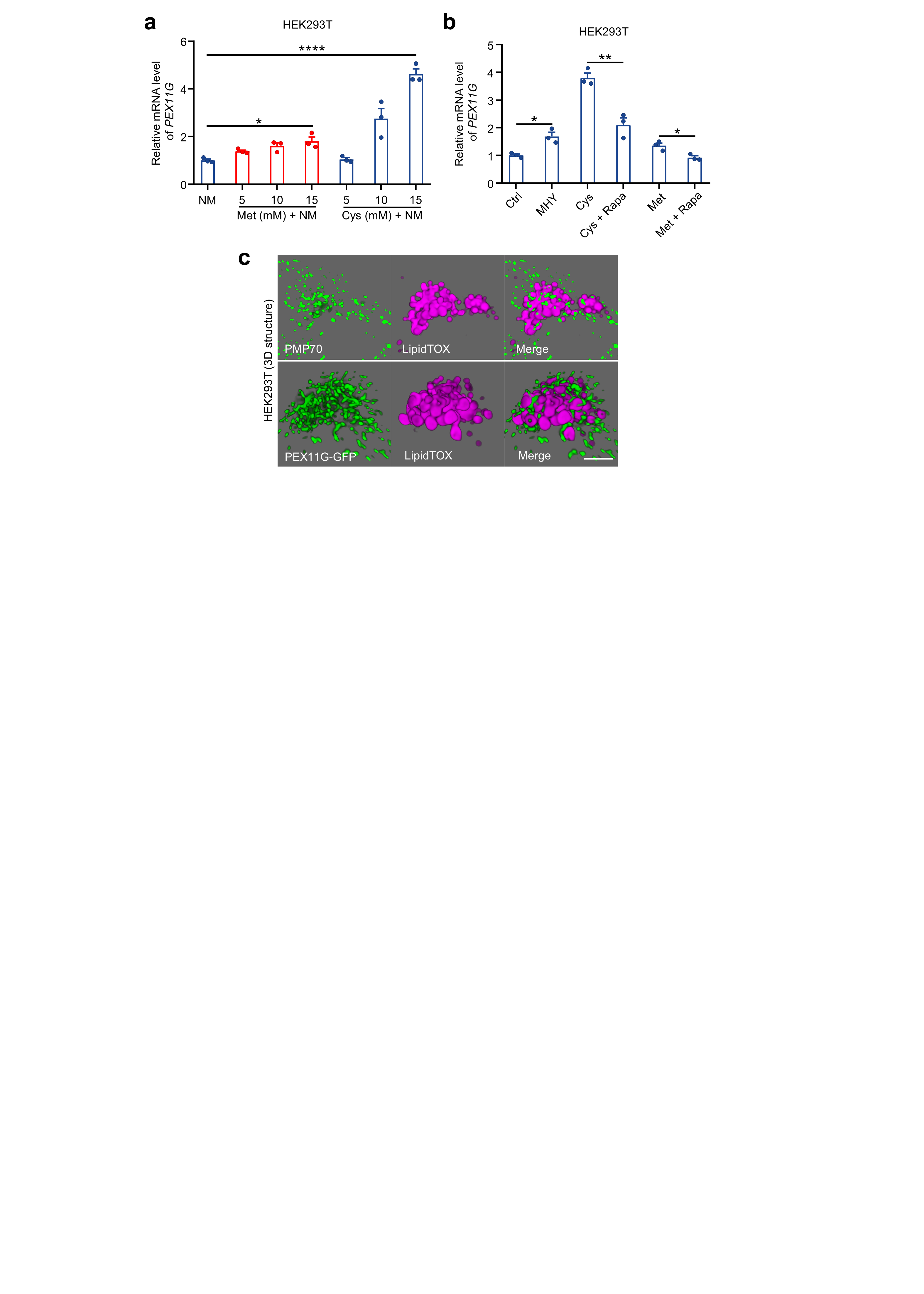
**Fig.S9 The functions of *PEX11G* are evolutionarily conserved.**

**a** Relative *PEX11G* mRNA levels in HEK293T cells cultured in the indicated media for 36 h. NM: normal medium; Met + NM: normal medium containing different concentrations of methionine; Cys + NM: normal medium containing different concentrations of cysteine. *n* = 3. **b** Relative *PEX11G* mRNA levels in HEK293T cells cultured in the indicated conditions for 36 h. Ctrl: normal medium; MHY: normal medium containing 2 μΜ MHY1485; Cys: normal medium containing 15 mΜ cysteine; Cys + Rapa: normal medium containing 15 mM cysteine and 500 nΜ rapamycin; Met: normal medium containing 15 mΜ methionine; Met + Rapa: normal medium containing 15 mM methionine and 500 nΜ rapamycin. *n* = 3. **c** Representative 3D structural images of peroxisome-LD contacts in HEK293T cells transfected with PEX11G-GFP (green) encoding plasmid or not and treated with 100 μΜ OA. LipidTOX (magenta) indicated neutral lipids. Scale bar, 5 μm. Two-tailed Student's *t* test was performed. * *p* < 0.05; ** *p* < 0.01; **** *p* < 0.0001.


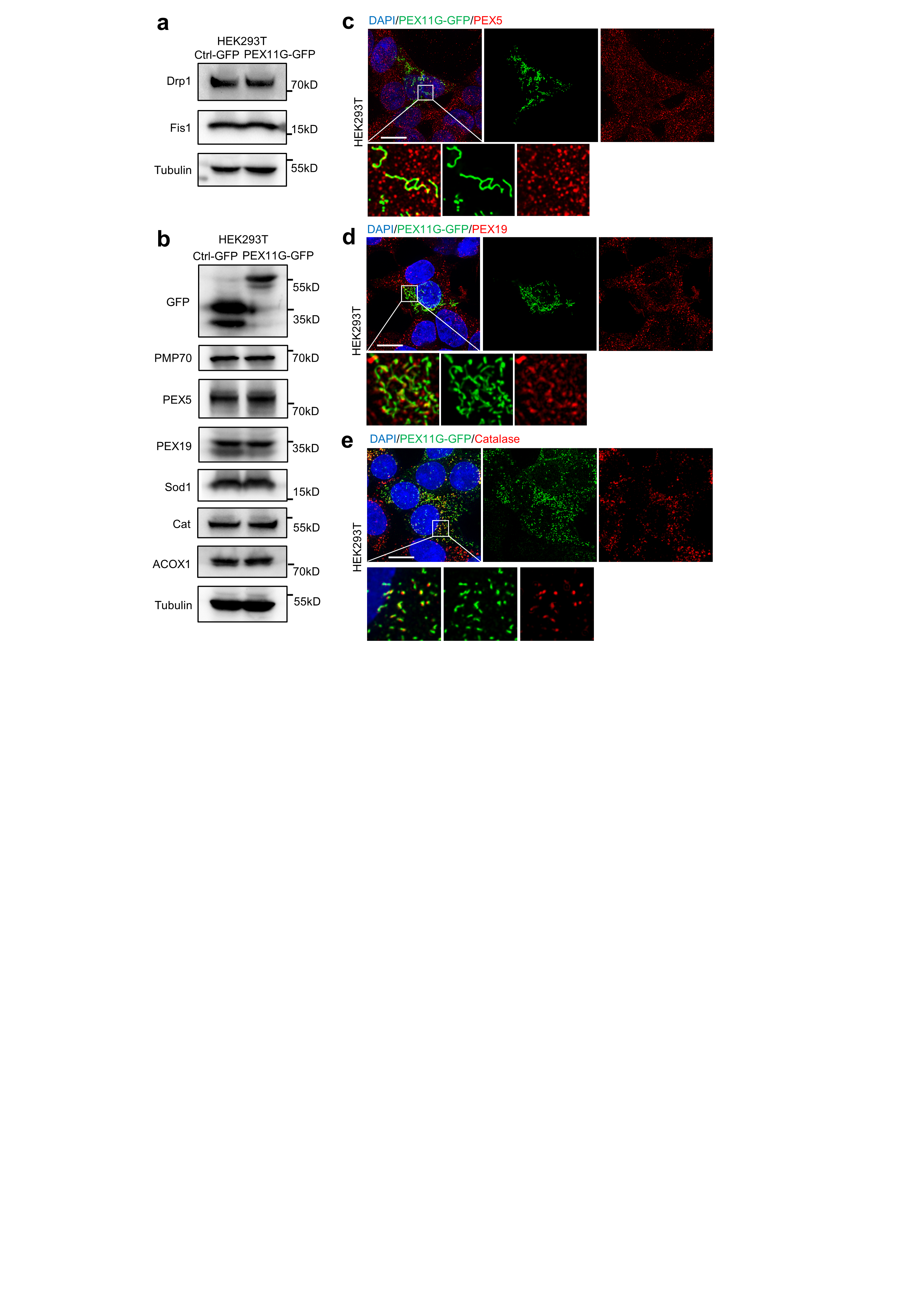
**Fig.S10** ***PEX11G* does not modulate the biosynthesis, import, or assembly of peroxisomes.**

**a** Western blotting showing the levels of Drp1 and Fis1 in HEK293T cells transfected with Ctrl-GFP or PEX11G-GFP encoding plasmids. **b** Western blotting showing the levels of GFP, PMP70, PEX5, PEX19, Sod1, Cat, and ACOX1 in HEK293T cells transfected with Ctrl-GFP or PEX11G-GFP encoding plasmids. **c-e** Fluorescence microscopy images of HEK293T cells transfected with PEX11G-GFP encoding plasmid. DAPI (blue) labeled nuclei. PEX5 (**c**), PEX19 (**d**), and Catalase (**e**) were stained in red. The boxed areas were enlarged to the lower panel. Scale bar, 20 μm.


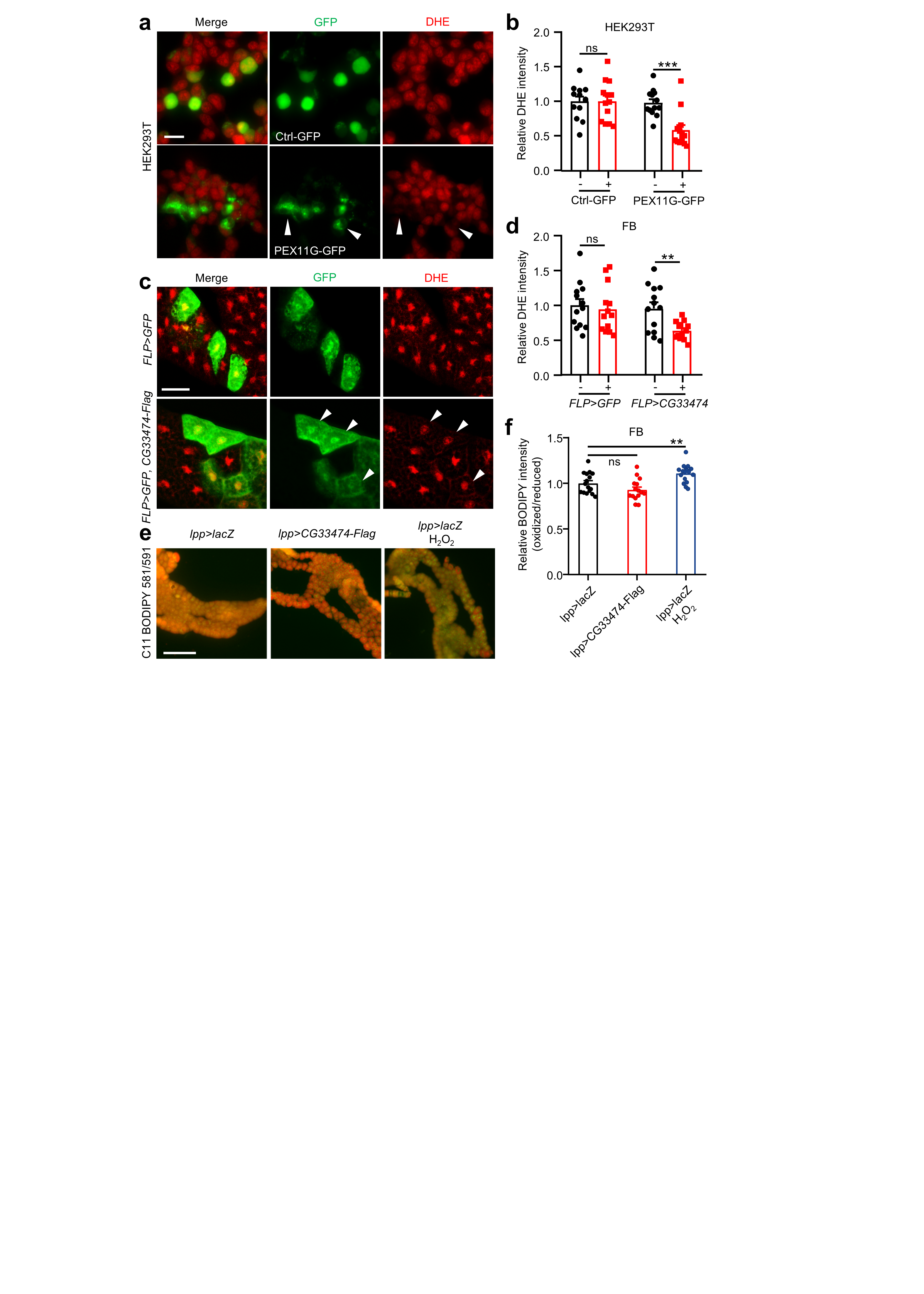
**Fig.S11 The functions of *PEX11G* are evolutionarily conserved.**

**a** Fluorescence microscopy images of HEK293T cells transfected with Ctrl-GFP (green) or PEX11G-GFP (green) encoding plasmids. DHE (red) indicated ROS levels. Arrows indicated cells that expressed *PEX11G*. Scale bar, 20 μm. **b** Relative fluorescence intensities of DHE in (**a**). **c** Evaluation of ROS levels in fat bodies from 3^rd^ instar larvae of the designated genotypes using DHE. Clones were labeled by GFP (green). DHE (red) indicated ROS levels. Arrows indicated fat cells that expressed *CG33474*. Scale bar, 50 μm. **d** Relative fluorescence intensities of DHE in (**c**). **e** Fluorescence microscopy images of fat bodies from the indicated genotypes stained with C11 BODIPY 581/591 probe. Larvae in the 3^rd^ columns were treated with 5% H_2_O_2_ for 36 h. Red: reduced form of C11-BODIPY; Green: oxidized form of C11-BODIPY. Scale bar, 200 μm. **f** Relative BODIPY intensities (oxidized/reduced) in (**e**). Two-tailed Student's *t* test was performed. ** *p* < 0.01; *** *p* < 0.001; ns, not significant.

**Table.S1** Primer sequences utilized in RT-qPCR (*Drosophila*).

| Gene name | Forward primers (5'-3') | Reverse primers (5'-3') |
| --- | --- | --- |
| *CG33474* | TTGATTCCTGTAGAGCCCGAG | GGTCTGGACTCTGATTGATTTGG |
| *Fis* | GTCTGGCTTAAAATACTGCCGA | CATACCCTTTGCCACTTCCTT |
| *Pex11ab* | ATCCTGAGCACATTCCGAAAAT | GCGTCACACGGATGTTAAGAT |
| *Pex11c* | ATTGTGCTACAGTGCCAAACT | CTCGCACCTGAGATCCTTGAG |
| *ABCD* | GCCTGACTAAACCATGCTTCG | CCCGATTGATAATGCTGGTCCA |
| *Pmp70* | CCGGCTCTAAGCAAATTGGC | GGTTTTCGCTTCTTGTTGGACAT |
| *CG31454* | ATCCCTGGCACATTTAAGACCT | TTTAGCGAAAGGAAAGCCGTC |
| *CG31259* | CTCTGAGATCCCTCCCAGTCT | CCTCCATTCATCGAAAGAAACGC |
| *CG11737* | CGCTGCTAGGGATCATCCAAT | GTGTAGAAGTTGTACCTCCCGA |
| *Pex3* | AGATGTGCCAAGCGGTTCTG | TCATGTCCTCCCAAAGTTCCAA |
| *Pex16* | AGACCACAGCCAAGTGGGTAT | CACCAGCATGTTTGACAGTGT |
| *Pex19* | TTGCTGGACAGTGCTCTCC | GGTCGCCACATCCGATGAT |
| *Pex1* | TTCAAACGCACTTTTAAGGTGGT | CCGTGTCATAGGTCGAAACGA |
| *Pex2* | CCGCCTGATACGCGATAATCT | GCTCCGGTTGGATTTTGATGAAC |
| *Pex5* | TGCGGTGGAGTTAATCCTCTC | GTCCATCTGAAATGACTGCGG |
| *Pex6* | ACAAGGGCAAGTTTCTGCTGA | AGGTGCTCCGACTTAATCTGC |
| *Pex7* | TCGAGGCTAACTACTTGCTGC | GGATGAGGAATTTGTGTTGGAGT |
| *Pex10* | CTCGGCGAAGAGTACACGG | CAGTTGTAACAATCTGCTGGGAA |
| *Pex12* | TTACCCGGCACTGAGCAAG | CCTCCTGAATCCTCAAACTCCC |
| *Pex13* | CGTATGGCAATGTTAGGGCTC | GTGTCTGCTGGAATGGGGAT |
| *Pex14* | GGTGCAGAACGATGTCGAGG | CTTTGGACCGTAGGAACTGCT |
| *RpL23* | GACAACACCGGAGCCAAGAACC | GACAACACCGGAGCCAAGAACC |

**Table.S2** Primer sequences utilized in RT-qPCR (HEK293T).

| Gene name | Forward primers (5'-3') | Reverse primers (5'-3') |
| --- | --- | --- |
| *Beta-actin* | GGCATGGGTCAGAAGGATTCCT | TCGTCCCAGTTGGTGACGAT |
| *PEX11G* | GGGGACACGTCTGTTGGTG | ACAGGGGTAGTAGAGCTGGTC |

**Video.S1** 3D visualization of fat bodies from *FLP>GFP-PTS1* 3rd instar larvae stained with Nile Red.

**Video.S2** 3D visualization of fat bodies from *FLP>GFP-PTS1, CG33474-Flag* 3rd instar larvae stained with Nile Red.

**Video.S3** 3D visualization of HEK293T cells transfected with Ctrl-GFP encoding plasmid.

**Video.S4** 3D visualization of HEK293T cells transfected with PEX11G-GFP encoding plasmid.

**Video.S5** Live cell image of U2OS cells transfected with PEX11G-GFP encoding plasmid.
